# A clustering-based competitive particle swarm optimization with grid ranking for multi-objective optimization problems

**DOI:** 10.1038/s41598-023-38529-4

**Published:** 2023-07-20

**Authors:** Qianlin Ye, Zheng Wang, Yanwei Zhao, Rui Dai, Fei Wu, Mengjiao Yu

**Affiliations:** 1 College of Computer Science and Technology, Zhejiang University of Technology, Hangzhou, 310023 China; 2 School of Computer and Computational Sciences, Hangzhou City University, Hangzhou, 310015 China; 3 School of Engineering, Hangzhou City University, Hangzhou, 310015 China

**Keywords:** Engineering, Electrical and electronic engineering

## Abstract

The goal of the multi-objective optimization algorithm is to quickly and accurately find a set of trade-off solutions. This paper develops a clustering-based competitive multi-objective particle swarm optimizer using the enhanced grid for solving multi-objective optimization problems, named EGC-CMOPSO. The enhanced grid mechanism involved in EGC-CMOPSO is designed to locate superior Pareto optimal solutions. Subsequently, a hierarchical-based clustering is established on the grid for improving the accuracy rate of the grid selection. Due to the adaptive division of clustering centers, EGC-CMOPSO is applicable for solving MOPs with various Pareto front (PF) shapes. Particularly, the inferior solutions are discarded and the leading particles are identified by the comprehensive ranking of particles in each cluster. Finally, the selected leading particles compete against each other, and the winner guides the update of the current particle. The proposed EGC-CMOPSO and the eight latest multi-objective optimization algorithms are performed on 21 test problems. The experimental results validate that the proposed EGC-CMOPSO is capable of handling multi-objective optimization problems (MOPs) and obtaining superior performance on both convergence and diversity.

## Introduction

When multiple objectives need to be optimized, they cannot all be optimal at the same time, resulting in contradictions between the objectives^1^. Such problems are referred to as MOPs. Assume for the sake of simplicity that a MOP consists of decision variables, $M$ objective functions, and certain constraints. The minimization of MOPs can be defined by the following mathematical expression:

1 $$\left\{ \begin{gathered} minf(x) = \left\{ {f_{1} (x),f_{2} (x), \ldots ,f_{M} (x)} \right\},x \in \Omega \hfill \\ g_{i} (x) \le 0,i = 1,2, \ldots ,p \hfill \\ h_{j} (x) = 0,j = 1,2, \ldots ,q \hfill \\ \end{gathered} \right.$$

where the decision variable $x = (x_{1} ,x_{2} , \ldots ,x_{D} ) \in \Omega$, the objective function $f(x) \in R^{M}$, $R^{D}$ denotes $D$-dimensional decision space, $R^{M}$ denotes M-dimensional objective space, there exists a map $\Omega \to R^{M}$, $g(x)$ is $p$ inequality constraints, and $h(x)$ is $q$ equality constraints.

Linear methods, unlike prior single-objective problems, cannot solve MOPs since there is no unique solution. Given their characteristics, researchers have proposed massive representative evolutionary algorithms (EAs) and swarm intelligence (SI) algorithms. Natural evolutionary selection drives the genetic algorithm (GA), and differential evolution (DE)^2^ is formed as a result of GA. SI is a branch of science that analyzes both natural and artificial systems. The ant colony optimization algorithm (ACO)^3^ is proposed after being inspired by ant foraging behavior. Similarly, particle swarm optimization (PSO)^4^ emerged from bird predation. The multi-objective evolutionary algorithm (MOEA)^5^ based on simulating chromosome crossing and mutation is a hot study topic among them. The Pareto solution set is obtained in these methods by optimizing many objectives under the given conditions. While the Pareto optimal solution set (PS) can achieve a compromise between convergence and diversity, that is, retain a uniform distribution of solutions while approaching PF indefinitely.

In PSO, the positions and velocities of particles are constantly updated based on individual global ideal positions ($gbest$) and historical optimal positions ($pbest$). The search efficiency can be effectively increased by interacting the information between the current particle and the optimal particle, and the population is steered to quickly converge to the true PF. The specific updated procedure is as follows:

2 $$v_{i} (t + 1) = \omega v_{i} (t) + c_{1} r_{1} (pbest_{i} (t) - x_{i} (t)) + c_{2} r_{2} (gbest_{i} (t) - x_{i} (t))$$

3 $$x_{i} (t + 1) = x_{i} (t) + v_{i} (t + 1)$$

where $x_{i} (t)$ and $v_{i} (t)$ denote the position and velocity of the $i$-th particle at $t$ iteration, respectively. $\omega$ represents an inertia weight factor, $c_{1}$ and $c_{2}$ are random acceleration factors greater than zero, $r_{1} ,r_{2} \in [0,1][0,1]$.

PSO has been successfully extended to multi-objective particle swarm optimization (MOPSO)^6^. Compared with MOEA, MOPSO converges more quickly. Existing MOPSO, on the other hand, is prone to local optima. The enhanced grid and clustering mechanism are introduced to address this issue. In summary, the primary contributions of EGC-CMOPSO are as follows:

1. The grid partition number is calculated dynamically based on the population size and the dimension of the objective space, and the coordinates of particles in the objective space are mapped to the grid space. Further, to maintain the particle variety, a novel enhanced grid ranking mechanism is developed.
2. This paper combines enhanced grid ranking with clustering. The bottom-up hierarchical clustering is adopted to construct clustering centers adaptively according to the particle distribution, avoiding the difficulties of artificial settings. Elite and leading particles are selected based on the normalized grid ranking value within each cluster and the distance to the cluster center.
3. Based on clustering and enhanced grid ranking, the competitive particle swarm optimization algorithm EGC-CMOPSO is proposed. The particle updating mechanism of traditional PSO has been improved, and no further external archiving is required. The winner of the leading particle guides the direct update of the current particle position and velocity, increasing computing efficiency.

The rest of this paper is organized as follows. Certain algorithms are introduced in Section “Related algorithms”. Section “Methods” describes our suggested EGC-CMOPSO in full. The experiment results of EGC-CMOPSO and the eight latest comparison algorithms on 21 test problems are discussed in Section “Results”. Finally, Section “Conclusion” makes a summary of this paper and put forward a prospect for the future.

## Related algorithms

### The existing MOPSO algorithms

The Pareto-domination-based approach is used in most MOPSOs to select leading particles. By integrating an adaptive grid mechanism and an adaptive mutation operator, Agrawal et al.^7^ proposed an interactive meta-heuristic multi-objective optimization (MOO) for roulette selection. Carvalho et al.^8^ coupled multi-objective control technique CDAS with PSO, and the proposed MOPSO-CDAS contributed to the improvement of convergence and solution diversity. To boost the convergence rate even further, Moubayed et al.^9^ integrated Pareto dominance and decomposition approaches into the solution mechanism. In D^2^MOPSO, a new technique for leading particle selection and external archive maintenance is proposed. The concept of the crowded distance between objective space and solution space is also introduced in it, which can fast converge without the genetic operator. A novel AgMOPSO^10^ directed by external archives is presented by Zhu et al., allocating relevant particles to each decomposed sub-problem for optimization. Due to the curse of dimensionality and poor performance in MOPs, the classical optimization algorithm is being considered. Lin et al.^11^ put forward a balanced approach to estimating fitness to solve this challenge and increase particle selection pressure. The novel speed update equation, simulated binary crossover (SBX) and evolutionary search based on polynomial variation (PM) are employed in NMPSO to considerably increase the algorithm’s performance.

Decomposition-based MOPSO decomposes MOPs into several sub-problems. Inspired by MOEA/D, Cain et al. proposed MPSO/D^12^, and the crowding distance is used in it to determine the fitness value to increase the convergence. A revolutionary multi-objective and multi-population co-evolution technique is proposed by Zhan et al., named CMPSO^13^. To improve the convergence rate, the elite information in the shared archive is utilized to adjust the particle speed. Meanwhile, the elite learning strategy is adopted to update the external archive to promote variety and avoid local optima. To meet multiple conflicting objectives, Yao et al.^14^ presented a co-evolutionary multi-group multi-objective optimization algorithm ECMSMOO. Furthermore, an endocrine incentive mechanism is embedded in it to avoid falling into local optimal. MMO-CLRPSO^15^ proposed by Zhang et al. uses a clustering strategy based on the Euclidean distance of the decision space for population division. Jiang et al.^16^ combined a convolutional neural network (CNN) with the decomposition strategy, and the proposed MOPSO/D-NET reconstructs NAS into a multi-objective evolutionary optimization problem.

In addition, the researchers increased selection pressure by introducing evaluation indicators. Common evaluation indexes include super-volume HV^17^, R2^18^, and related MOPSO including MaOPSO/vPF^19^ and IDMOPSO^20^.

### The grid mechanism

**Figure 1:**
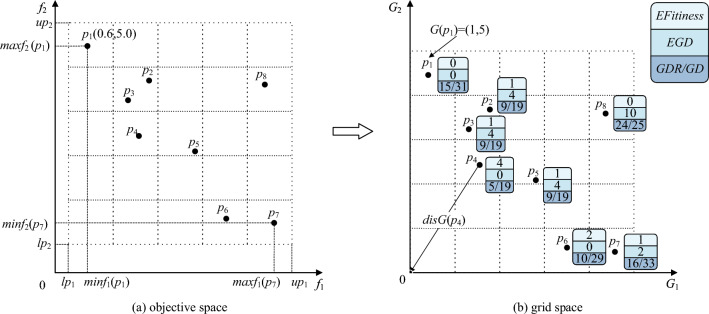
The mapping of objective space to grid space.

$gbest$ is not the only option available to lead the evolution direction of the population in MOPs. The grid mechanism is first incorporated into MOPs by Knowles et al.^21^, thus ensuring the diversity of non-inferior solutions in external archiving. Suppose to select promising particles in the external archive, take Fig. 1a as an example and try to select particles in different grids, thus, $p_{2}$ and $p_{3}$ cannot be selected simultaneously. The diversity of particles can be visualized by mapping grid coordinates, and convergence can be measured by the dominance of the grid. Based on these advantages, Numerous grid-based optimization algorithms have emerged, e.g., GrEA^22^, MOPSO^23^ and GSMPSO-MM^24^, etc. We improve the traditional grid mechanism in EGC-CMOPSO, drawing inspiration from GrEA, and the enhanced grid ranking provides a more comprehensive evaluation of the convergence and diversity of particles. Under the premise of uniform distribution, $EGD$ and the ratio of $GDR$, $GD$ determine $p_{1}$, $p_{4}$, $p_{6}$ and $p_{7}$ can converge to the PF more quickly in Fig. 1b. Keep in mind that in EGC-CMOPSO, the number of the grid varies dynamically with population size and dimension. The larger the size of the population, the smaller the grid size, and the more significant the population diversity.

## Methods

### The framework of EGC-CMOPSO

The framework of the proposed EGC-CMOPSO is presented in Algorithm 1.
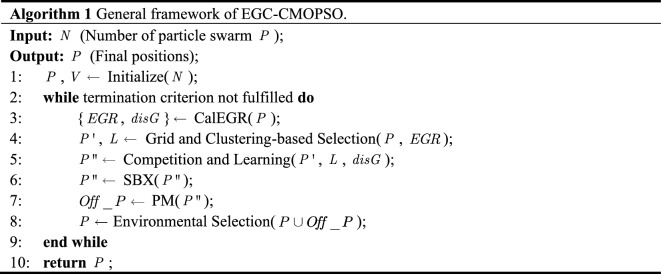


Prior to the iteration, firstly, randomly initialize the velocities and positions of $N$ particles (line 1). To obtain more diverse particles in the optimization process, utilize CalEGR (line 3) to enhance the original grid sorting to get $EGR$ and $disG$. Then, adopt GC (line 4) to generate the promising particles ($P^{\prime}$). This novel selection strategy is performed to pick out the leading particle set ($L$) with better comprehensive ranking values to guide the following competition learning. Next, similar to the competition-based learning method adopted by CMOPSO^25^, employ CL (line 5) to find the winning particle ($p_{win}$) to direct the update of the current particle. After that, use both SBX and PM (lines 6–7) to generate offspring ($Off\_P$). Finally, the environmental selection mechanism (line 8) in SPEA2^26^ is used directly to select $N$ better particles for the next generation.

### Enhanced grid ranking

Traditionally, the Pareto dominance relation is used to select elite particles, but in the late iteration, the selection pressure is insufficient, leading to the concentration of most particles in the first Pareto front surface. The introduction of the grid concept can improve the diversity and convergence of the population effectively by sorting particles in the grid coordinate system. Taking advantage of the grid mechanism, enhanced grid ordering is proposed in this paper, and the strategy is described in detail below.

#### Definition 1

(*Partition number of grid coordinates*). The grid is utilized in EGC-CMOPSO to relocate the position of particles in the objective space, which is transformed from the decision space. The design of $div$ is critical in the iterative process of the population. Taking into account the impact of $N$ and $M$, and $div$ is changed adaptively as follows:

4 $$div = \frac{\sqrt N }{{\log M}}$$

where $N$ denotes the number of particles, $M$ denotes the number of objectives. Apparently, with the change of $N$ and $M$, the partition number of grid coordinates is also adjusted adaptively. Increasing or decreasing the selection pressure dynamically allows for improved convergence to the true PF, effectively improving the convergence and diversity of the final population.

GrEA is the source for grid-related definitions. The upper and lower boundaries of the grid on the $i$-th objective can be determined:

5 $$lp_{i} = maxf_{i} (x) + \frac{{maxf_{i} (x) - minf_{i} (x)}}{2 \times div}$$

6 $$up_{i} = maxf_{i} (x) + \frac{{maxf_{i} (x) - minf_{i} (x)}}{2 \times div}$$

where $div$ represents the partition number of grid coordinates on every dimension, $minf_{i} (x)$ and $maxf_{i} (x)$ represent the minimum and maximum values of $x$ on the $i$-th objective, respectively.

#### Definition 2

(*Grid coordinates*). Based on the above definition of $div$, $up$ and $lp$, the grid coordinate corresponding to the particle $x$ in the objective space is determined by:

7 $$G_{i} (x) = \left\lfloor {div \times \frac{{f_{i} (x) - lp_{i} }}{{up_{i} - lp_{i} }}} \right\rfloor + 1$$

where $G_{i} (x)$ denotes the grid coordinate of $i$-th ($i = 1,2 \ldots ,M$) objective of the particle $x$, $f_{i} (x)$ is the actual value of the particle $x$ on the $i$-th objective and $\left\lfloor . \right\rfloor$ represents the floor function. For example, in Fig. 1a, the coordinate of the particle $p_{1}$ is ${(0}{\text{.6,5}}{.0)}$, after $p_{1}$ is mapped to grid space in Fig. 1b, $G(p_{1} ) = (1,5)$. As a result, the essence of grid-based dominance is similar to Pareto dominance, consequently, $x$ grid-dominates $y$ ($y \succ_{grid} x$) can be defined as:

8 $$\begin{gathered} \forall i \in (1,2, \ldots ,M):G_{i} (x) \le G_{i} (y) \wedge \hfill \\ \exists j \in (1,2, \ldots ,M):G_{j} (x) < G_{j} (y) \hfill \\ \end{gathered}$$

To make the selection of an elite particle set more reasonable, that is, particles close to the Preto boundary are selected under the premise of ensuring the diversity of particles. Inspired by this, we propose three evaluation indexes based on grid coordinates, namely enhanced grid dominance ($EGD$), grid dominance ranking ($GDR$), and grid difference ($GD$). Among them, $EGD$ and $GDR$ are used to evaluate the convergence of particles, while $GD$ is used to evaluate the diversity of particles, and $EGR$ combines the above three indexes effectively.

#### Definition 3

(*Enhanced fitness values*). Based on the above concept of grid domination, the enhanced fitness value of each particle in the grid coordinate system is evaluated by constructing the accumulation function of the dominant particles. $EFitness$ is an index of convergence and lays a certain foundation for the subsequent judgment of enhanced grid domination. Specifically, each particle is assigned a $EFitness$, representing the number of grid-dominate particles, denoted as follows:

9 $$EFitness(x) = \left| {\left\{ {y|y \in P \wedge y \succ_{grid} x} \right\}} \right|$$

where $\left| . \right|$ denotes the number of elements in a set that meet the given conditions.

#### Definition 4

(*Enhanced grid dominance*). Based on $EFitness$, $EGD$ can be calculated directly:

10 $$EGD(x) = \sum\limits_{{y \in P,x \succ_{grid} y}} E Fitness(y)$$

According to the definition, $EGD$ is also a convergence index. Instead of merely examining the domination connection between particles, $EGD$ is determined jointly by the set of particles dominated by the grid and the number of particles it dominates. The smaller $EGD$, the better, i.e., $EGD(x) = 0$ implies that no other particle can overpower it. That is, $x$ is a non-inferior solution. Similarly, a higher $EGD(x)$ means existing many particles $y$ grid dominates $x$, and $y$ dominates many particles at the same time, resulting in a larger sorted value of $x$ in our proposed algorithm.

#### Definition 5

(*Grid dominance ranking*). $GDR$ is used to evaluate the convergence of particles. The sum of grid-dominated coordinate differences on all objectives is calculated as follows:

11 $$GDR(x_{i} ) = \sum\limits_{{x_{i} \ne x_{j} }} {\sum\limits_{m = 1}^{M} {\max } } \left( {G_{m} \left( {x_{i} } \right) - G_{m} \left( {x_{j} } \right),0} \right)$$

#### Definition 6

(*Grid difference assignment*). Different from the $EGD$ and $GDR$ indicators of convergence defined previously, the $GD$ index focuses on the representation of particle diversity, and the sum of the absolute grid coordinate differences on each objective is defined as follows:

12 $$GD(x_{i} ) = \sum\limits_{{x_{i} \ne x_{j} }} {\sum\limits_{m = 1}^{M} {\left| {G_{m} \left( {x_{i} } \right) - G_{m} \left( {x_{j} } \right)} \right|} }$$

The larger the $GD$ value, the better the diversity and the more uniform particles are distributed.

#### Definition 7

(*Enhanced grid ranking*). We can generate an index $EGR$ that incorporates diversity and convergence fully by combining the three measures mentioned above:

13 $$EGR = \frac{\ln (EGD + 1)}{{\ln 2}} + \frac{GDR}{{GD}}$$

From the above definition, it is clear that $EGD$, $GDR$ and $GD$ are used to calculate $EGR$. Based on this, Algorithm 2 is proposed to realize the calculation of $disG$ (lines 3–5) and $EGR$ (lines 6–9). Finally, take them as a return to guide the selection of $L$ (line 5 in Algorithm 1).
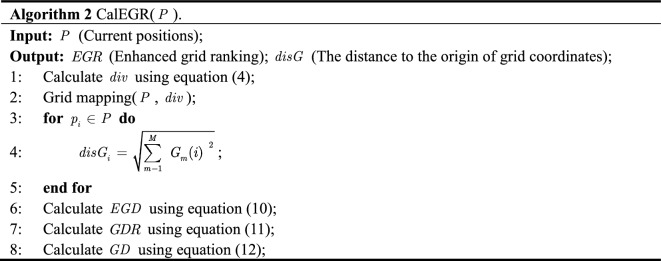


Although the three indices of $EGD$, $GDR$ and $GD$ balance convergence and diversity well, considering the values of $GDR$ and $GD$ maybe the same, and the number of particles is an integer, this results in the same $GDR$. As shown in Fig. 1b, particles $p_{2}$, $p_{3}$ and $p_{5}$ have the same $EGR = 2.80$, and the quality of these particles cannot be discriminated adequately in the grid. To solve this problem, the concept of hierarchical clustering is introduced in the following.

### Hierarchical clustering strategy

**Figure 2:**
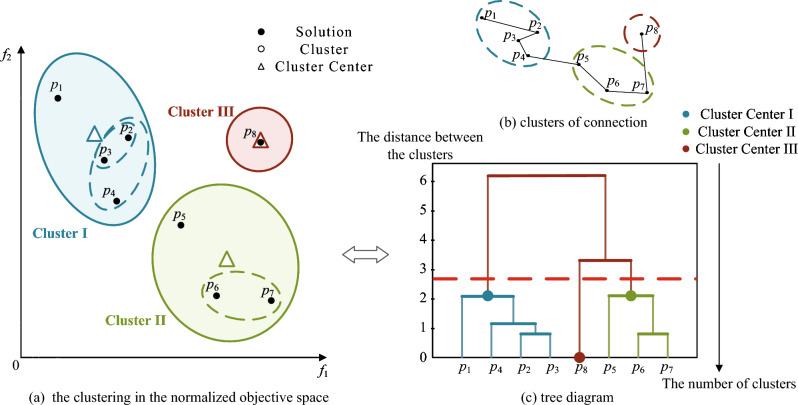
Illustration of the bottom-up hierarchical clustering.


In the field of MOPs, CA-MOEA^27^ demonstrates the feasibility and superiority of hierarchical clustering, especially for MOPs with irregular PF. Considering that the positions of non-dominated solutions constantly change at different iteration stages, the clustering centers are generated adaptively based on the positions of particles, and the populations are dynamically divided. Then, combined with the previously proposed $EGD$ in each cluster to reorder particles, eliminate the poor particles after reordering, and select the leading particles required for the next step. The implementation process of this strategy is shown in Fig. 2. Algorithm 3 depicts the specific implementation.

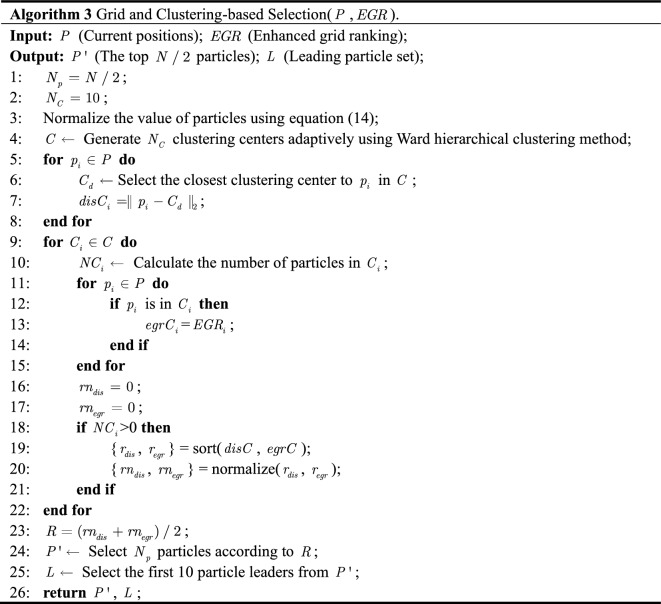


To make the objective values of each dimensional particle comparable, the values of the particle on each objective are normalized to [0,1] before clustering (line 2), taking into account the varying effects of different dimensions on the final results:

14 $$z_{i,nor} = \frac{{z_{i} - z_{i,\min } }}{{z_{i,\max } - z_{i,\min } }}$$

where $z_{i,nor}$ represents the corresponding objective value after $z_{i}$ is normalized, $z_{i}$ denotes the $i$-th objective value of the particle, $z_{i,\min }$ and $z_{i,\max }$ denote the minimum and maximum value on the $i$-th objective of the particle $z_{i}$, respectively.

Next, particles are assigned to the nearest cluster center ($C_{d}$) based on the Euclidean distance between each cluster center. Iterate through each cluster center again, and calculate the number of particles assigned to the cluster (lines 4–7). The number of particles assigned to the cluster is counted (line 9) by traversing each cluster center. If no particle in the cluster, the sorting value of the particle closest to the distance from the cluster center is assigned to 0 (lines 15–16). If there are particles in the cluster, sort them according to the Euclidean distance to the clustering center ($C_{i}$) and $EGR$ inside the cluster, to get $rn_{dis}$ and $rn_{egr}$ (lines 17–20). Since the two sorting values initially correspond to different objects, to make them comparable, the two sorting values before comprehensive sorting are normalized (line 19). Finally, according to the combination ranking value based on grid and clustering, the top ten are selected as the leading particles ($L$), and $N/2$ promising particles ($P^{\prime}$) are selected for the next competitive learning (lines 22–25).

**Figure 3:**
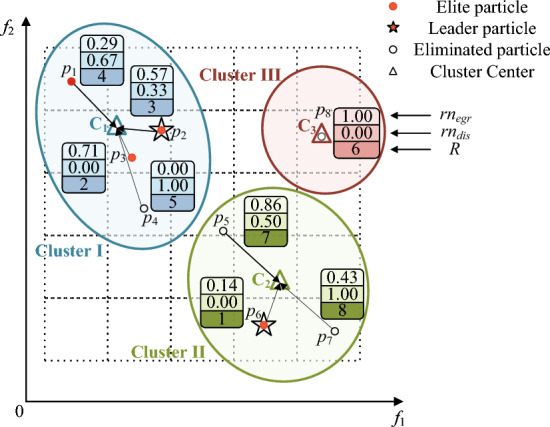
Illustration of clustering-based selection with grid ranking.

For simplicity, Fig. 3 is taken as an example. The comprehensive ranking values can be derived by integrating the normalized ranking: $R(p_{5} ) = 7$, $R(p_{6} ) = 1$ and $R(p_{7} ) = 8$. That is, $p_{6}$ ranks first in the comprehensive ranking of the eight particles in the figure. Therefore, in Fig. 3, $p_{2}$ and $p_{6}$ with the first and second $R$ values are selected as $L$. The top four $R$ values, $p_{1}$, $p_{2}$, $p_{3}$ and $p_{6}$ are retained for the next competitive learning, because of the inclusion of more information on convergence and diversity.

It is worth noting that the size of $L$ affects the convergence rate of particles, to a lesser extent, the convergence of the final solution set to a certain extent. The size of $L$ sensitivity analysis is tested on the DTLZ1-3 and DTLZ7 in CMOPSO. Thereby, the size of $L$ is set to 10 in our proposed algorithm.

### Competitive PSO

The basic idea of competitive PSO is composed of two parts: determining the winning particle and guiding the update by the winner. $p_{win}$ is determined by comparison with the current particles in the elite particle set ($P^{\prime}$). It is worth noting that, different from the classical PSO, no traditional external archiving mechanism is adopted in EGC-CMOPSO. Each update operation of the current particle $p_{i}$ is directly determined by $p_{win}$. As a result, no additional computational resources and space are required to calculate and store local and global optimal particles. Algorithm 4 provides the pseudo-code of the competitive PSO mechanism.
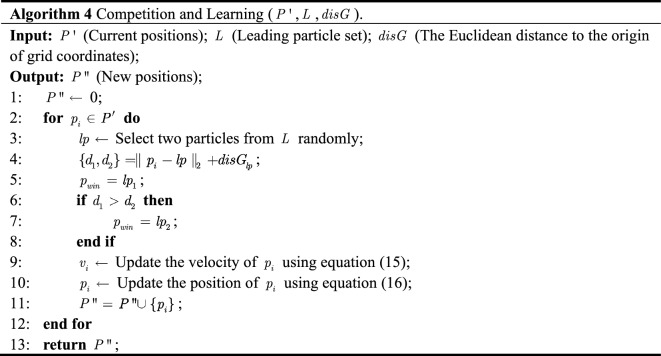


**Figure 4:**
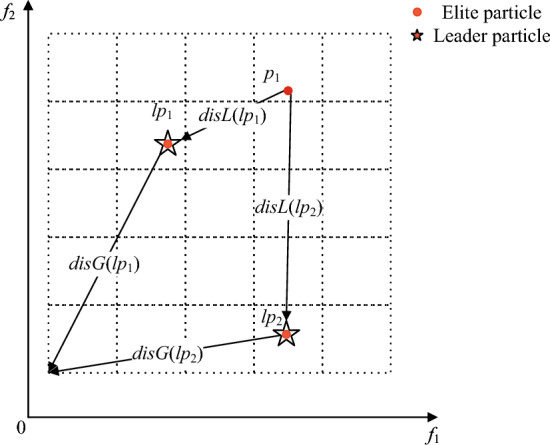
The selection mechanism of winning particles.

The first part is to select the winner particle. Two particles are randomly selected as competitors from $L$ (line 3), e.g., particles $lp_{1}$ and $lp_{2}$ in Fig. 4. A particle in the elite particle set $p_{i} \in P^{\prime}$ is taken as the particle to be updated, e.g., the particle $p_{1}$ in Fig. 4. The Euclidean distances of $lp_{1}$, $lp_{2}$ to $p_{i}$ and the origin of the grid are calculated, respectively (line 4). The leading particle with the smallest combined distance is selected as $p_{win}$ (lines 5–8) to guide the update of $p_{1}$.

The second part is the update with the guidance of the winning particle. The winner directs the update of the position and velocity of the current particle. Because the competition-based particle learning mechanism considers just the winner, not $pbest$ and $gbest$, the traditional formulation for updating position and speed is updated:

15 $$v_{i} (t + 1) = r_{1} v_{i} (t) + r_{2} (x_{win} (t) - x_{i} (t))) \,$$

16 $$x_{i} (t + 1) = x_{i} (t) + v_{i} (t + 1) + r_{1} (v_{i} (t + 1) - v_{i} (t))) \,$$

where $v_{i} (t + 1)$ and $x_{i} (t + 1)$ represent the speed and position of the $i$-th particle at ($t + 1$) iteration, namely, the speed and position after $p_{i}$ competition update, $r_{1}$ and $r_{2}$ are two randomly generated vectors with values between 0 and 1, $x_{win}$ denotes the position of $p_{win}$.

### Environmental selection

The generated offspring and the primary population are taken as input, and the optimal solution set is selected by the mechanism of environmental selection. For simplicity, the environmental selection mechanism in SPEA2 is used in ER-MOPSO to select $N$ promising particles for the next generation. SPEA2’s truncation strategy sequentially eliminates individuals with minimum distance from their neighbors, which is an effective method to promote population convergence and diversity.

### Computational complexity analysis

The algorithm is mainly determined by the calculation of $EGR$, selection based on grid and clustering, and competitive learning. In the first part, the calculation of $EGR$ (line 4 in Algorithm 1) requires $O(MN^{2} )$. In the second part, the time complexity of bottom-up hierarchical clustering (line 3 in Algorithm 3) is $O(MN^{2} )$. Allocating $N$ particles to the corresponding cluster center (lines 4–7 in Algorithm 3) requires $O(MN)$, and the calculation of normalized sorting values based on grid and clustering (lines 8–21 in Algorithm 3) is of complexity $O(MN\log N)$. Therefore, the overall time complexity of algorithm 3 is $O(MN^{2} )$. In the third part, the competitive particle swarm optimizer (lines 2–12 in Algorithm 4) requires $O(MN)$. In addition, the worst-case complexity of truncation operations in environment selection is $O(N^{3} )$. In conclusion, the overall computational complexity of EGC-CMOPSO in each generation is the maximum in $O(MN^{2} )$ and $O(N^{3} )$, which is equivalent to NSGA-II, CA-MOEA and PREA.

## Results

### Experimental settings

This section verifies the good performance of our proposed EGC-CMOPSO in convergence and diversity. To evaluate the performance of EGC-CMOPSO to handle complex MOPs with variously shaped PFs, we carried out a series of simulation experiments on DTLZ1–DTLZ7, WFG1–WFG9, ZDT1-–ZDT4^28^ and ZDT6, which have irregular PFs. Under the same experimental platform PlatEMO^29^, EGC-CMOPSO was compared with eight state-of-the-art multi-objective optimization algorithms: NSGA-II + ARSBX^30^, CA-MOEA, MOEA/D-CMA^31^ PREA^32^, Two_Arch2^33^, GrEA, NMPSO and CMOPSO.

To quantify the performance of EGC-CMOPSO quantitatively, four frequently used performance metrics are used, namely, IGD^34^, HV, Spacing^35^, and Spread^36^. Supplementary methods online provide extra information on the experimental setup.

The operating environment of the algorithms is in Matlab R2021a, and all comparison algorithms run on a PC with Intel Core i9-10900 K, CPU 3.70 GHz and RAM 64.00 GB. The following experiments are carried out on the PlatEMO platform to ensure the consistency of the experimental setting.

The Wilcoxon rank sum test is adopted to compare the results of the algorithms intuitively. The best performance metric in all comparison algorithms on the corresponding column test problems is marked in bold. The symbols “+”, “−” and “=” indicate the results obtained by the comparison algorithms are significantly better, worse and approximate to EGC-CMOPSO, respectively, at the significance level of 0.05. On the ZDT3 test problem, for example, the fourth row in Table 1 indicates that the IGD values of the different comparison algorithms are worse than our proposed algorithm.

**Table 1:** IGD values of EGC-CMOPSO and eight comparison algorithms. significant values are in bold.

Problem	NSGAIIARSBX	CAMOEA	MOEADCMA	PREA	Two_Arch2	GrEA	NMPSO	CMOPSO	EGCCMOPSO
ZDT1	4.6531e−3 (1.98e−4)−	4.5335e−3 (1.36e−4)−	3.8994e−3 (1.09e−5)=	4.2888e−3 (1.57e−4)−	**3.8092e−3 (3.08e−5)+**	7.3423e−3 (1.10e−3)−	2.8706e−2 (1.06e−2)−	4.1138e−3 (7.60e−5)−	3.9120e−3 (4.89e−5)
ZDT2	4.7307e−3 (1.63e−4)−	4.4199e−3 (1.14e−4)−	**3.8224e−3 (7.68e−6)+**	4.4551e−3 (1.59e−4)−	4.7349e−3 (1.87e−5)−	7.9473e−3 (1.22e−4)−	1.9972e−2 (4.17e−3)−	4.0752e−3 (7.02e−5)−	3.8787e−3 (3.86e−5)
ZDT3	5.2867e−3 (1.90e−4)−	5.1109e−3 (1.66e−4)−	1.0836e−2 (3.63e−5)−	5.9553e−3 (5.36e−3)−	6.3968e−3 (7.46e−3)−	1.4024e−2 (9.93e−4)−	1.0095e−1 (8.10e−4)−	4.6589e−3 (6.74e−5)−	**4.5705e−3 (4.83e−5)**
ZDT4	4.5486e−3 (1.97e−4)−	4.3805e−3 (1.82e−4)−	3.8983e−3 (7.43e−6)−	4.3147e−3 (1.61e−4)−	3.9319e−3 (6.45e−5)−	3.6131e−1 (1.45e−1)−	2.9867e−2 (1.58e−2)−	**3.8398e−3 (2.96e−5)+**	3.8743e−3 (3.87e−5)
ZDT6	3.7233e−3 (1.36e−4)−	3.4295e−3 (8.64e−5)−	3.1044e−3 (6.37e−6)−	3.4157e−3 (7.25e−5)−	3.3587e−3 (3.61e−5)−	6.1647e−3 (2.07e−5)−	4.4733e−3 (4.83e−4)−	3.7399e−3 (1.39e−4)−	**3.0709e−3 (1.75e−5)**
DTLZ1	2.6243e−2 (1.18e−3)+	2.1397e−2 (3.89e−4)+	**2.0687e−2 (1.18e−4)+**	2.1866e−2 (5.35e−4)+	2.1096e−2 (2.97e−4)+	6.9568e+0 (2.06e+0)−	2.2874e−2 (1.43e−3)+	3.2460e+0 (4.50e+0)=	2.4716e+0 (3.46e+0)
DTLZ2	6.9491e−2 (3.32e−3)−	5.7071e−2 (8.98e−4)−	5.5529e−2 (4.34e−4)−	5.7401e−2 (7.02e−4)−	5.8928e−2 (1.61e−3)−	5.4954e−2 (7.12e−4)=	7.6973e−2 (3.41e−3)−	6.7091e−2 (1.94e−3)−	**5.4808e−2 (5.68e−4)**
DTLZ3	6.8843e−2 (3.69e−3)+	5.8967e−2 (3.07e−3)+	1.9424e+0 (6.49e+0)+	**5.7279e−2 (8.72e−4)+**	5.8048e−2 (1.84e−3)+	2.7332e+1 (6.55e+0)+	7.6408e−2 (2.17e−3)+	5.2557e+1 (3.13e+1)+	8.1506e+1 (2.91e+1)
DTLZ4	6.7679e−2 (2.45e−3)−	5.7335e−2 (7.50e−4)−	1.1531e−1 (8.04e−2)−	3.5310e−1 (3.27e−1)−	8.6455e−2 (1.62e−1)=	7.1384e−2 (8.87e−2)−	7.6722e−2 (2.62e−3)−	1.2859e−1 (2.22e−1)−	**5.6342e−2 (8.29e−4)**
DTLZ5	5.8597e−3 (3.44e−4)−	5.1122e−3 (1.67e−4)−	2.2768e−2 (6.95e−5)−	4.6739e−3 (1.35e−4)−	5.8903e−3 (3.47e−4)−	6.8862e−3 (5.52e−4)−	1.5234e−2 (2.87e−3)−	6.1616e−3 (3.68e−4)−	**4.2846e−3 (6.85e−5)**
DTLZ6	5.6115e−3 (3.29e−4)−	4.6541e−3 (1.26e−4)−	2.2885e−2 (2.37e−5)−	4.6779e−3 (1.36e−4)−	6.2492e−3 (1.46e−5)−	1.1328e−1 (2.48e−2)−	1.4716e−2 (3.48e−3)−	5.3144e−3 (2.52e−4)−	**4.1797e−3 (4.54e−5)**
DTLZ7	7.8262e−2 (3.97e−3)−	8.0415e−2 (7.31e−2)−	1.5133e−1 (5.19e−3)−	2.2530e−1 (2.61e−1)=	6.6276e−2 (5.26e−2)−	9.3116e−2 (5.47e−2)−	6.9417e−2 (4.10e−3)−	1.3865e−1 (1.48e−1)−	**5.9279e−2 (1.11e−3)**
WFG1	2.2995e−1 (1.26e−2)−	1.6666e−1 (6.40e−3)=	4.4221e−1 (1.03e−1)−	1.6064e−1 (4.68e−3)+	**1.3892e−1 (2.96e−3)+**	1.7872e−1 (5.57e−3)−	5.7320e−1 (2.23e−1)−	1.1708e+0 (1.48e−1)−	1.6903e−1 (2.21e−2)
WFG2	2.1489e−1 (1.02e−2)−	1.8171e−1 (6.01e−3)−	2.4414e−1 (1.80e−2)−	1.7557e−1 (5.65e−3)=	**1.5490e−1 (3.28e−3)+**	2.2835e−1 (1.25e−2)−	4.5097e−1 (5.36e−2)−	2.1323e−1 (1.15e−2)−	1.7509e−1 (5.28e−3)
WFG3	8.8850e−2 (1.50e−2)+	1.4234e−1 (1.23e−2)−	1.3753e−1 (3.23e−3)−	8.1256e−2 (7.24e−3)+	7.8758e−2 (4.42e−3)+	9.7682e−2 (5.01e−3)+	**4.2503e−2 (3.10e−3)+**	1.0580e−1 (2.02e−2)+	1.2652e−1 (8.56e−3)
WFG4	2.9248e−1 (1.19e−2)−	2.3211e−1 (3.85e−3)−	3.3374e−1 (1.62e−2)−	2.2678e−1 (3.61e−3)−	2.2439e−1 (4.93e−3)−	2.4697e−1 (3.18e−3)−	3.0795e−1 (1.19e−2)−	3.0258e−1 (9.03e−3)−	**2.2122e−1 (3.19e−3)**
WFG5	2.8400e−1 (1.33e−2)−	2.3928e−1 (2.68e−3)−	2.9660e−1 (9.95e−3)−	2.3454e−1 (3.64e−3)−	2.3454e−1 (4.76e−3)−	2.5519e−1 (2.63e−3)−	2.9702e−1 (9.99e−3)−	2.8219e−1 (1.00e−2)−	**2.2340e−1 (2.13e−3)**
WFG6	3.0213e−1 (1.12e−2)−	2.6058e−1 (9.51e−3)−	3.1159e−1 (3.04e−2)−	2.4541e−1 (9.68e−3)−	2.4640e−1 (1.10e−2)−	2.6526e−1 (7.94e−3)−	4.0612e−1 (9.45e−3)−	2.8598e−1 (1.47e−2)−	**2.2486e−1 (7.01e−3)**
WFG7	2.8636e−1 (1.07e−2)−	2.3433e−1 (5.30e−3)−	2.7430e−1 (1.95e−2)−	2.2897e−1 (3.55e−3)−	2.2269e−1 (4.62e−3)−	2.4860e−1 (1.50e−3)−	3.0892e−1 (1.36e−2)−	2.7030e−1 (8.77e−3)−	**2.1478e−1 (3.01e−3)**
WFG8	3.8214e−1 (1.15e−2)−	3.2555e−1 (6.62e−3)−	3.6561e−1 (6.28e−3)−	**2.8273e−1 (4.85e−3)+**	2.9884e−1 (6.46e−3)+	2.9239e−1 (5.76e−3)+	3.4821e−1 (1.12e−2)−	3.8795e−1 (1.15e−2)−	3.0771e−1 (4.47e−3)
WFG9	2.6774e−1 (9.56e−3)−	2.3130e−1 (4.08e−3)−	2.9791e−1 (5.76e−3)−	2.2529e−1 (3.63e−3)−	**2.2141e−1 (5.01e−3)+**	2.3981e−1 (4.09e−3)−	3.3511e−1 (4.94e−2)−	2.6060e−1 (8.10e−3)−	2.2201e−1 (3.43e−2)
+/−/=	3/18/0	2/18/1	3/17/1	5/14/2	8/12/1	3/17/1	3/18/0	3/17/1	

### Performance comparison

The mean and standard deviation of IGD values of EGC-CMOPSO and other comparison algorithms are represented in Table 1. For the universality of the algorithm considerations, the results of 21 test problems with two or three objectives are listed. Each row provides a horizontal comparison of the results of the ERC-CMOPSO and comparison algorithms on a particular problem, where the best mean of all algorithms in 30 independent runs is highlighted against a grey background. The longitudinal comparison of IGD values of ERC-CMOPSO on different test problems is shown in each column.

EGC-CMOPSO outperforms the comparison algorithms in most cases, yielding 11 optimal outcomes on 21 test problems, particularly on the DTLZ test suite. Hierarchical clustering is used in both EGC-CMOPSO and CA-MOEA, but EGC-CMOPSO performs better in 18 test problems compared to CA-MOEA. The reason for the different results may lie in the introduction of the grid mechanism based on clustering, to select particles with better overall performance to guide competition, indicating the necessity of the grid mechanism. Similarly, EGC-CMOPSO is more competitive than GrEA in 17 test problems. This is attributed to the fact that GrEA only considers grid advantages and has certain limitations in some special cases. While clustering-based with grid ranking is adopted in the proposed EGC-CMOPSO, with better competitiveness. PREA is a newly proposed evolutionary algorithm, that performs worse than EGC-CMOPSO on 14 test problems. Compare to NSGA-II + ARSBX, MOEA/D-CMA, Two_Arch2, NMPSO, and CMOPSO, EGC-CMOPSO shows its competitiveness on most test problems. Similarly, the experimental results of HV, SP and Spread metrics are shown in Supplementary Tables S3–S5 online, respectively.

**Figure 5:**
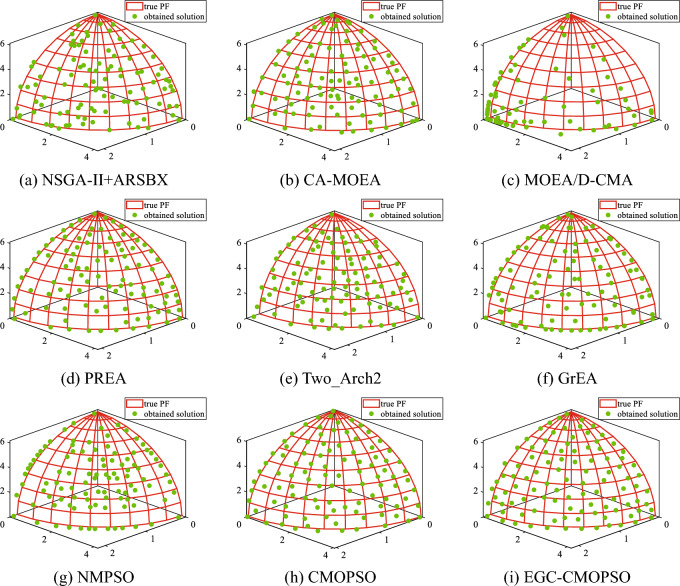
The non-dominated solutions obtained by EGC-CMOPSO and eight comparison algorithms on three-objective WFG6.

The distributions of the non-dominated solutions are depicted in Supplementary Figs. S1, S2, and Fig. 5. Supplementary Fig. S1 shows the Pareto fronts obtained by nine algorithms and the theoretical Pareto front after 50 iterations on the ZDT3 problem. Clearly, the solutions obtained by EGC-CMOPSO are most consistent with real PF, and the distribution is uniform on the real PF. While the convergence and distribution of the solutions obtained by NMPSO are the worst, this may be due to too few iterations. Similarly, it can be seen from Supplementary Fig. S2 that EGC-CMOPSO is superior to other comparison algorithms on the irregular three-objective DTLZ6. In contrast, the solution distributions of CA-MOEA, MOEA/D-CMA and PREA have poor convergence, maybe because the convergence speed of the evolutionary algorithm is not as fast as PSO in the early stage. Due to the competitive learning mechanism, CMOPSO performs poorly when compared to other PSO algorithms. This is possibly due to the fact that the winning particle guiding particle updating may not be the optimal particle, and hence cannot guide particles to quickly get closer to real PF at the beginning of the iteration. For the three-objective WFG6, EGC-CMOPSO is shown to be competitive in Fig. 5. While most of the solutions obtained by the algorithms converge to PF, the solutions obtained by EGC-CMOPSO are more uniformly distributed.

The PFs obtained from Fig. 5 show that the algorithms have largely converged after 50 iterations. Therefore, we compared the time required for all algorithms to iterate 50 times on test problems with different PFs. The experimental results in Table S6 of the supplementary material reveal that the clustering operation increases the computational burden of EGC-CMOPSO and the time required for iteration becomes larger, which is a drawback of our proposed algorithm.

To investigate the effect of population size on the performance of the algorithm, $N$ = 100, 200 and 500 are set to be tested under 9 typical problems against the eight latest algorithms, respectively. IGD is selected as the comprehensive evaluation index and Spread is the diversity rating index for the experiments. The box line plots for nine algorithms under various evaluation indexes and population sizes are shown in Supplementary Figs. S3–S8 online.

### Comparison on convergence

To test the performance of EGC-CMOPSO on two-objective and three-objective problems, the index change curves of EGC-CMOPSO and eight comparison algorithms on ZDT3 and DTLZ6 are shown in Supplementary Figs. S9 and S10 online, respectively. Our algorithm is illustrated by a curve with a red five-pointed star among them. As shown in the figures, IGD, SP and Spread of EGC-CMOPSO are the smallest among all comparison algorithms in most cases. Similarly, taking Fig. S10b as an example, with the increase in iteration times, the HV value of EGC-CMOPSO always remains optimal or suboptimal.

It is worth mentioning that, the convergence rate of EGC-CMOPSO is significantly faster than other algorithms prior to 50 generations. This indicates that EGC-CMOPSO inherits the advantages of PSO with fast convergence speed in the early stage, and based on it, integrates enhanced grid and clustering mechanisms to maintain particle diversity well.

### Influence of the number of clusters

In EGC-CMOPSO, the number of clusters ($N_{c}$) and grids ($div$) are introduced to implement clustering-based with enhanced grid ranking. To explore the sensitivity of EGC-CMOPSO performance to these two parameters, two related experiments are designed respectively.

The performance metrics of EGC-CMOPSO with different numbers of clusters are shown in Supplementary Figs. S11 and S12 online. In the experiment, the population size is fixed at 100, the calculated resource is fixed at 1000 × $N$. $N_{c}$ is set in ascending order to 2, 10, 20, 50 and 100. To test the performance of EGC-CMOPSO on various challenges, the experiments are conducted on 4 two-objective and 7 three-objective test problems, respectively. In summary, when $N_{c}$ = 10, each performance indicator on different test problems is better. Too large $N_{c}$ results in too few particles in the cluster and affect the sorting selection. While too small $N_{c}$ results in too many particles in the cluster, which cannot make good use of the advantages of clustering. Therefore, $N_{c}$ is set to 10 in EGC-CMOPSO is the best option.

### Influence of the number of grids

**Figure 6:**
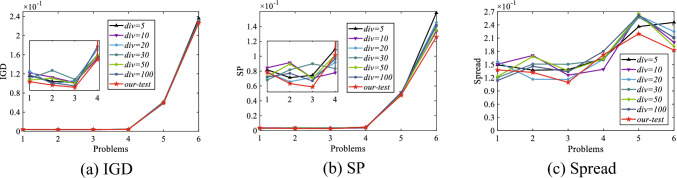
IGD, SP and Spread of EGC-CMOPSO with the different numbers of grids on 6 test problems. The values on the x-coordinate denote the following test problems: 1 = ZDT1, 2 = ZDT2, 3 = ZDT4, 4 = DTLZ6, 5 = DTLZ7, 6 = WFG7.

In studies to investigate the sensitivity of the number of grids, the size of the population is kept constant at 100, the computational resource is fixed as 1000 × $N$, and the number of iterations is 50. $div$ is set to 5, 10, 20, 30, 50 and 100, the adaptive value of $div$ obtained by using Eq. (4). The calculation of $div$ used in EGC-CMOPSO is represented by a curve with a red five-pointed star. As evidenced by the results in Fig. 6, in most cases, the div obtained by Eq. (4) makes EGC-CMOPSO has better competitiveness on ZDT1, ZDT2, ZDT4, DTLZ6, DTLZ7 and WFG7. Therefore, it is feasible to use our custom $div$ in EGC-CMOPSO.

## Conclusion

This paper proposes a clustering-based competitive particle swarm optimization with the enhanced grid ranking algorithm. The grid of EGC-CMOPSO is constructed in objective space, and bottom-up hierarchical clustering is utilized to generate clustering centers adaptively. Each particle is assigned to the corresponding cluster. To assist in locating the leading particles inside each cluster, the normalized enhanced grid ranking is fused to conduct a comprehensive sorting for particles. The current particle is updated by the competitive particle swarm optimization based on the winner selected from the leading particles each time. Different from the other PSOs, the external archiving mechanism is not adopted in EGC-CMOPSO. Compared with the eight latest algorithms, EGC-CMOPSO is competitive on test problems with regular or irregular PF, and the obtained solutions strike a good balance of convergence and diversity.

Given the increasing complexity of MOPs, our future research direction is to apply the clustering-based grid ranking mechanism to high-dimensional, large-scale, complex optimization problems with constraints. Furthermore, complicated MOPs in the real world deserve to be investigated, since EGC-CMOPSO has demonstrated its competitiveness in addressing MOPs.

## Supplementary Information

Supplementary Information.

## Data Availability

The datasets used during the study are available from the corresponding author upon reasonable request.
