# Supplementary material for: A clustering-based competitive particle swarm optimization with grid ranking for multi-objective optimization problems
Source: Sci Rep. 2023 Jul 20;13:11754. doi: 10.1038/s41598-023-38529-4 (PMC10359354; doi:10.1038/s41598-023-38529-4)
Supplement: Supplementary file 1 — Supplementary Information. [file 41598_2023_38529_MOESM1_ESM.pdf]

A clustering-based competitive particle swarm optimization with grid ranking for multi-objective optimization problems

Supplementary Methods

Considering the PF of some complex MOPs is not regular, DTLZ1-DTLZ7, WFG1-WFG9, ZDT1-ZDT4 and ZDT6 are used to test the performance of EGC-CMOPSO, In the simulation experiment, we set the dimension of decision variables $n=30$ for ZDT1-ZDT3, $n=10$ for ZDT4 and ZDT6 for the two-objective problem. For the three-objective problem, according to the different dimensions of the objective, the dimension of decision variables of DTLZ and WFG test suites are set as $n=M+k-1$, where $k=5$ in DTLZ1, $k=20$ in DTLZ7, $k=10$ in DTLZ2-DTLZ6 and WFG1-WFG9. A variety of linear, convex, concave and disconnected Pareto front shapes are contained in the ZDT, WFG and DTLZ test suites. Among them, ZDT1, ZDT3, ZDT4, DTLZ7, and WFG1-WFG3 have irregular PFs, which can be used for evaluation and to evaluate the ability of EGC-CMOPSO to handle complex MOPs with PF of different shapes. Significantly, the number of position-related parameters k of the WFG must be divisible by the number of underlying position parameters. In summary, the specific characteristics of all benchmark functions used in the experiment are listed in Table SI.

To verify the performance of EGC-CMOPSO, we choose eight state-of-the-art algorithms as comparison algorithms: NSGA-II+ARSBX, CA-MOEA, MOEA/D-CMA, PREA, Two_Arch2, GrEA, NMPSO and CMOPSO. The parameter settings of comparison algorithms are summarized in Table S2. For a fair comparison, these parameter settings are recommended by their authors. The population size (N) is set to 100 in our experiment, and the maximum number of iterations is set to 10,000. To ensure comparability, the external archive set size in the above algorithms is set to equal N . Each algorithm is run 30 times on each test problem independently.

Further, to be fair to the comparison, the parameters of the comparison algorithms are set according to the original literature. Taking an example of GrEA, the number of grid divisions is set to div=45 in the two-objective problem and div=15 in the three-objective problem. While the div in EGC-CMOPSO is computed using formula4. In addition, the crossover probability of SBX p_c is set to 1.0, and the distribution index N_c is set to 20. Similarly, the mutation probability p_m is set to $1/\text{n}$, and the distribution index N_m is set to 20 in PM.

Different evaluation mechanisms of different indicators lead to different emphases of algorithm performance evaluation.

1) Inverted Generational Distance (IGD): To evaluate the convergence of the algorithm, Generational Distance (GD) is calculated by the average Euclidean distance of each solution to a point on the nearest real Pareto front (PF_{true}). In consideration of GD cannot evaluate the convergence and diversity of the algorithm at the same time, IGD is proposed to solve this problem, the average distance between each reference point on PF_{true} and the nearest solution in the solution set is calculated. Since the solutions on PF_{true} are uniformly distributed, the solution set obtained by the algorithm converges to PF_{true} and is evenly distributed, by starting from PF_{true}. The more sampling points, the more uniform distribution, and the more accurate and reliable the results. IGD is a comprehensive indicator, and its computational complexity is $O(M\,|P||P'|)$.
2) Hypervolume (HV): A given PF is needed in the computation of GD and IGD, while only a reference point is needed in HV to evaluate the quality of the perspectives of convergence and distribution. HV is a general comprehensive index, and the time complexity of it is $O(|P'|^{M-1})$.
3) Spacing (SP): SP is a diversity indicator used to evaluate the distribution of solutions. The smaller SP is, the more evenly distributed the solution is. The square of the difference between the two nearest solutions and their average is calculated. The time complexity of SP is $O(M\,|P'|^2)$.
4) Spread (Δ): The extent of the spread measure tactic is proposed in Δ. As a diversity indicator,

the smaller Δ is, the more dispersed the solution set is and the better the expansibility is. The time complexity of Δ is $O(M \mid P \mid\mid P' \mid)$.

Supplementary Table

Table S1. The characteristics of benchmark functions.

Problem	M	n	k	PF shape
ZDT1	2	30	-	convex
ZDT2	2	30	-	concave
ZDT3	2	30	-	disconnected
ZDT4	2	10	-	convex
ZDT6	2	10	-	concave
DTLZ1	3	M=1+k	5	linear
DTLZ2	3	M=1+k	10	concave
DTLZ3	3	M=1+k	10	concave
DTLZ4	3	M=1+k	10	concave
DTLZ5	3	M=1+k	10	concave
DTLZ6	3	M=1+k	10	concave
DTLZ7	3	M=1+k	10	disconnected
WFG1	3	M-1+k	10	convex, mixed
WFG2	3	M-1+k	10	convex
WFG3	3	M-1+k	10	disconnected
WFG4	3	M-1+k	10	linear, degenerate
WFG5	3	M-1+k	10	concave
WFG6	3	M-1+k	10	concave
WFG7	3	M-1+k	10	concave
WFG8	3	M-1+k	10	concave
WFG9	3	M-1+k	10	concave

Table S2. The parameter settings for comparison algorithms.

Algorithems	Parameter settings
NSGA-II+ARSBX	$N=100$, $p_s=0.5$, $p_c=1.0$, $N_c=20$, $p_m=1/n$, $N_m=20$
CA-MOEA	$N=100$, $p_c=1.0$, $N_c=20$, $p_m=1/n$, $N_m=20$
MOEA/D-CMA	$N=100$, $T=N/10$, $K=5$, $p_m \in (0,1)$, $\eta \in Z^+$
PREA	$N=100$, $p_s=0.7$, $p_c=1.0$, $N_c=20$, $p_m=1/n$, $N_m=20$
Two_Arch2	$N=100$, CAsize=N, $p=1/M$, $p_c=1.0$, $N_c=20$, $p_m=1/n$, $N_m=20$
GrEA	$N=100$, div=45,15 for $M=2,3$, $p_c=1.0$, $N_c=20$, $p_m=1/n$, $N_m=20$
NMPSO	$N=100$, $\omega \in [0.1, 0.5]$, $c_1,c_2,c_3 \in [1.5,2.5]$, $p_m=1/n$, $N_m=20$
CMOPSO	$N=100$, $\gamma=10$, R_1, $R_2 \in [0,1]$, $p_m=1/n$, $N_m=20$
EGC-CMOPSO	$N=100$, $p_c=1.0$, $N_c=20$, $p_m=1/n$, $N_m=20$

Table S3. HV values of EGC-CMOPSO and eight comparison algorithms.

Problem	NSGAIIARSBX	CAMOEA	MOEADCMA	PREA	Two_Arch2	GrEA	NMPSO	CMOPSO	EGCCMOPSO
ZDT1	7.1937e-1	7.1964e-1	7.2009e-1	7.2006e-1	7.2036e-1	7.1540e-1	6.9044e-1	7.1957e-1	7.2041e-1
	(2.45e-4) -	(1.31e-4) -	(6.99e-5) -	(1.59e-4) -	(1.82e-6) -	(1.33e-3) -	(1.29e-2) -	(1.85e-4) -	(7.32e-5)
ZDT2	4.4413e-1	4.4436e-1	4.4478e-1	4.4457e-1	4.4496e-1	4.4154e-1	4.3518e-1	4.4417e-1	4.4499e-1
	(2.23e-4) -	(1.49e-4) -	(7.02e-5) -	(1.40e-4) -	(2.54e-6) -	(5.91e-5) -	(2.59e-3) -	(2.12e-4) -	(5.75e-5)
ZDT3	5.9947e-1	5.9893e-1	5.9802e-1	6.0213e-1	6.0553e-1	5.9725e-1	5.6688e-1	5.9970e-1	5.9973e-1
	(8.39e-5) -	(3.43e-4) -	(1.90e-4) -	(1.62e-2) +	(2.26e-2) +	(3.72e-4) -	(3.20e-4) -	(5.02e-5) -	(4.36e-5)
ZDT4	7.1979e-1	7.1971e-1	7.2009e-1	7.1994e-1	7.2007e-1	4.9486e-1	6.8807e-1	7.2038e-1	7.2054e-1
	(1.85e-4) -	(3.74e-4) -	(6.12e-5) -	(1.93e-4) -	(2.33e-4) -	(1.00e-1) -	(1.84e-2) -	(1.02e-4) -	(2.86e-5)
ZDT6	3.8826e-1	3.8855e-1	3.8888e-1	3.8858e-1	3.8865e-1	3.8586e-1	3.8772e-1	3.8828e-1	3.8890e-1
	(1.37e-4) -	(7.80e-5) -	(1.39e-4) =	(7.35e-5) -	(2.08e-5) -	(2.30e-5) -	(4.29e-4) -	(1.18e-4) -	(2.70e-5)
DTLZ1	8.2568e-1	8.3784e-1	8.4034e-1	8.4025e-1	8.4080e-1	0.0000e+0	8.3229e-1	4.8628e-2	8.7359e-2
	(3.52e-3) +	(1.31e-3) +	(6.36e-4) +	(7.71e-4) +	(3.65e-4) +	(0.00e+0) -	(4.57e-3) +	(1.57e-1) =	(2.17e-1)
DTLZ2	5.2854e-1	5.5087e-1	5.5637e-1	5.5918e-1	5.6009e-1	5.5402e-1	5.6061e-1	5.2744e-1	5.5214e-1
	(4.20e-3) -	(2.34e-3) -	(3.69e-4) +	(1.06e-3) +	(9.19e-4) +	(1.92e-3) +	(1.14e-3) +	(4.93e-3) -	(1.99e-3)
DTLZ3	5.3159e-1	5.5057e-1	3.6094e-1	5.5804e-1	5.5946e-1	0.0000e+0	5.6089e-1	0.0000e+0	0.0000e+0
	(6.28e-3) +	(3.86e-3) +	(2.61e-1) +	(1.92e-3) +	(1.97e-3) +	(0.00e+0) =	(1.81e-3) +	(0.00e+0) =	(0.00e+0)
DTLZ4	5.3119e-1	5.5159e-1	5.3745e-1	4.1931e-1	5.4493e-1	5.4608e-1	5.6159e-1	4.9609e-1	5.4811e-1
	(2.93e-3) -	(1.73e-3) +	(2.97e-2) -	(1.65e-1) =	(8.58e-2) -	(3.80e-2) -	(9.52e-4) +	(1.10e-1) -	(1.70e-3)
DTLZ5	1.9870e-1	1.9914e-1	1.9041e-1	1.9975e-1	2.0017e-1	1.9746e-1	1.9550e-1	1.9853e-1	1.9994e-1
	(3.77e-4) -	(1.49e-4) -	(3.22e-5) -	(1.41e-4) -	(5.43e-5) +	(5.10e-4) -	(8.98e-4) -	(2.46e-4) -	(8.93e-5)
DTLZ6	1.9959e-1	1.9985e-1	1.9047e-1	1.9985e-1	2.0034e-1	1.2967e-1	1.9602e-1	1.9973e-1	2.0016e-1
	(1.35e-4) -	(1.09e-4) -	(7.90e-6) -	(1.25e-4) -	(1.63e-5) +	(2.40e-2) -	(1.03e-3) -	(1.65e-4) -	(3.04e-5)
DTLZ7	2.6737e-1	2.7186e-1	2.5882e-1	2.5791e-1	2.7785e-1	2.7646e-1	2.7554e-1	2.5852e-1	2.7822e-1
	(1.99e-3) -	(8.96e-3) -	(7.05e-4) -	(2.70e-2) -	(6.40e-3) -	(9.66e-4) -	(1.09e-3) -	(1.39e-2) -	(7.44e-4)
WFG1	9.2460e-1	9.4202e-1	8.5898e-1	9.4769e-1	9.4688e-1	8.7642e-1	8.1266e-1	4.4884e-1	9.3841e-1
	(5.15e-3) -	(1.03e-3) =	(3.89e-2) -	(5.96e-4) +	(1.02e-3) +	(2.74e-2) -	(6.88e-2) -	(6.08e-2) -	(1.37e-2)
WFG2	9.1913e-1	9.2941e-1	9.0255e-1	9.3644e-1	9.3537e-1	9.2497e-1	8.6214e-1	9.1556e-1	9.2912e-1
	(2.76e-3) -	(1.54e-3) =	(1.52e-2) -	(8.07e-4) +	(9.87e-4) +	(1.84e-3) -	(2.12e-2) -	(2.24e-3) -	(2.06e-3)
WFG3	4.0081e-1	3.6525e-1	3.7342e-1	3.9887e-1	4.0007e-1	3.1121e-1	4.0905e-1	3.9365e-1	3.7188e-1
	(2.76e-3) +	(7.09e-3) -	(2.02e-3) =	(2.73e-3) +	(1.83e-3) +	(5.93e-2) -	(2.17e-3) +	(4.77e-3) +	(4.51e-3)
WFG4	5.0509e-1	5.3648e-1	5.0273e-1	5.5951e-1	5.5787e-1	5.3422e-1	5.5842e-1	4.8434e-1	5.3756e-1
	(4.97e-3) -	(2.34e-3) =	(1.67e-2) -	(9.92e-4) +	(9.24e-4) +	(3.79e-3) -	(1.16e-3) +	(6.09e-3) -	(3.77e-3)
WFG5	4.7945e-1	5.0964e-1	4.6729e-1	5.2024e-1	5.1825e-1	5.1064e-1	5.2064e-1	4.7614e-1	5.0543e-1
	(1.05e-2) -	(2.04e-3) +	(8.69e-3) -	(1.01e-3) +	(8.21e-4) +	(2.20e-3) +	(1.05e-3) +	(7.06e-3) -	(2.39e-3)
WFG6	4.9470e-1	4.9168e-1	4.9246e-1	5.1046e-1	5.1187e-1	4.9489e-1	4.1741e-1	5.0157e-1	5.3036e-1
	(8.38e-3) -	(1.10e-2) -	(2.92e-2) -	(1.40e-2) -	(1.45e-2) -	(1.30e-2) -	(1.32e-3) -	(1.16e-2) -	(1.19e-2)
WFG7	5.1258e-1	5.3787e-1	5.2981e-1	5.5989e-1	5.5801e-1	5.3485e-1	5.5966e-1	5.1522e-1	5.5186e-1
	(5.67e-3) -	(2.18e-3) -	(1.59e-2) -	(7.96e-4) +	(1.15e-3) +	(3.10e-3) -	(9.73e-4) +	(3.71e-3) -	(1.31e-3)
WFG8	4.2224e-1	4.4887e-1	4.1509e-1	4.8203e-1	4.7735e-1	4.4674e-1	4.8443e-1	4.1896e-1	4.5266e-1
	(5.25e-3) -	(2.57e-3) -	(6.27e-3) -	(1.83e-3) +	(2.02e-3) +	(3.92e-3) -	(1.85e-3) +	(4.68e-3) -	(3.14e-3)
WFG9	4.9866e-1	5.1424e-1	4.8085e-1	5.5278e-1	5.4019e-1	5.1205e-1	4.9071e-1	5.0307e-1	5.1585e-1
	(5.29e-3) -	(3.46e-3) -	(7.07e-3) -	(2.51e-3) +	(3.72e-3) +	(5.09e-3) -	(6.57e-2) =	(4.19e-3) -	(3.07e-2)
+/-/=	3/18/0	4/14/3	3/16/2	12/8/1	14/7/0	2/18/1	9/11/1	1/18/2	

Table S4. SP values of EGC-CMOPSO and eight comparison algorithms.

Problem	NSGAIIARSBX	CAMOEA	MOEADCMA	PREA	Two_Arch2	GrEA	NMPSO	CMOPSO	EGCCMOPSO
ZDT1	6.4621e-3	5.4821e-3	9.9735e-3	4.7958e-3	6.9339e-3	1.1104e-2	4.5095e-2	3.0672e-3	3.1817e-3
	(6.95e-4) -	(4.54e-4) -	(5.21e-5) -	(5.83e-4) -	(1.26e-4) -	(2.34e-3) -	(1.07e-2) -	(3.30e-4) =	(2.89e-4)
ZDT2	6.7484e-3	5.4841e-3	4.2338e-3	4.8846e-3	6.8826e-3	1.0039e-2	3.2488e-2	3.1438e-3	3.1809e-3
	(6.76e-4) -	(3.98e-4) -	(2.05e-5) -	(4.96e-4) -	(1.25e-4) -	(5.28e-4) -	(5.33e-3) -	(2.94e-4) =	(2.73e-4)
ZDT3	7.6024e-3	6.0607e-3	2.3446e-2	5.8699e-3	3.8294e-3	1.2853e-2	1.0760e-1	3.4682e-3	3.1809e-3
	(8.48e-4) -	(6.07e-4) -	(1.78e-4) -	(2.65e-3) -	(3.70e-4) -	(1.29e-3) -	(4.31e-4) -	(3.31e-4) -	(3.74e-4)
ZDT4	7.4337e-3	5.4795e-3	9.9449e-3	4.7123e-3	6.3951e-3	5.2772e-1	4.4747e-2	2.6659e-3	3.1981e-3
	(8.21e-4) -	(5.58e-4) -	(7.26e-5) -	(5.55e-4) -	(3.68e-4) -	(8.54e-1) -	(1.30e-2) -	(2.59e-4) +	(3.16e-4)
ZDT6	6.1560e-3	4.5042e-3	7.3525e-3	3.8029e-3	8.6904e-3	5.5205e-3	8.2362e-3	3.2564e-2	1.2040e-2
	(6.49e-4) +	(3.58e-4) +	(2.47e-2) +	(4.48e-4) +	(2.29e-2) +	(2.13e-4) +	(1.76e-3) +	(7.60e-2) -	(4.14e-2)
DTLZ1	2.0893e-2	1.7177e-2	2.3251e-3	1.1329e-2	1.2933e-2	1.0908e+1	2.1646e-2	3.0288e-1	1.4715e-1
	(1.52e-3) +	(1.66e-3) +	(8.11e-4) +	(1.41e-3) +	(1.27e-3) +	(8.53e+0) -	(4.85e-3) +	(4.01e-1) -	(3.61e-1)
DTLZ2	5.7552e-2	4.4323e-2	6.2731e-2	2.8905e-2	4.0477e-2	2.3877e-2	5.1534e-2	5.2918e-2	2.4675e-2
	(5.77e-3) -	(2.94e-3) -	(2.80e-3) -	(2.90e-3) -	(3.74e-3) -	(2.36e-3) =	(4.11e-3) -	(4.55e-3) -	(1.86e-3)
DTLZ3	1.1362e+0	2.2503e+0	3.2097e-1	3.0132e-2	5.1150e-1	4.7148e+1	5.1216e-2	5.0769e+0	7.9156e+0
	(3.48e+0) +	(6.23e+0) +	(8.29e-1) +	(2.84e-3) +	(1.69e+0) +	(3.52e+1) -	(4.12e-3) +	(4.25e+0) +	(5.20e+0)
DTLZ4	5.7956e-2	4.4615e-2	7.1846e-2	1.5713e-2	3.5581e-2	2.3940e-2	5.0637e-2	5.2307e-2	2.4163e-2
	(5.73e-3) -	(2.22e-3) -	(2.76e-2) -	(1.28e-2) =	(7.60e-3) -	(4.32e-3) =	(4.68e-3) -	(1.49e-2) -	(2.36e-3)
DTLZ5	8.8704e-3	7.6035e-3	1.3087e-2	5.7670e-3	9.0426e-3	5.8582e-3	2.9199e-2	8.5588e-3	4.2908e-3
	(9.71e-4) -	(4.33e-4) -	(4.34e-3) -	(7.62e-4) -	(8.53e-4) -	(7.94e-4) -	(4.02e-3) -	(8.97e-4) -	(3.94e-4)
DTLZ6	1.0654e-2	6.8784e-3	7.0602e-3	6.1632e-3	1.1430e-2	1.9672e-1	2.8955e-2	8.6792e-3	4.3609e-3
	(1.10e-3) -	(5.25e-4) -	(1.38e-3) -	(7.29e-4) -	(1.10e-4) -	(1.23e-1) -	(4.37e-3) -	(7.48e-4) -	(3.92e-4)
DTLZ7	7.4491e-2	5.5099e-2	1.3073e-1	3.4060e-2	3.7315e-2	6.9917e-2	7.2029e-2	6.8082e-2	4.8615e-2
	(7.72e-3) -	(8.51e-3) -	(4.24e-3) -	(1.18e-2) +	(5.48e-3) +	(1.11e-2) -	(8.05e-3) -	(1.48e-2) -	(3.96e-3)
WFG1	1.6375e-1	1.7299e-1	1.8814e-1	1.2374e-1	2.1731e-1	1.5920e-1	5.3191e-1	2.7925e-1	2.8176e-1
	(1.37e-2) +	(6.63e-2) +	(3.56e-2) +	(1.96e-2) +	(2.65e-2) +	(1.43e-2) +	(4.42e-2) -	(5.94e-2) =	(1.63e-2)
WFG2	2.3713e-1	1.8089e-1	2.5929e-1	1.3857e-1	1.3951e-1	1.4489e-1	3.2197e-1	2.2242e-1	1.5368e-1
	(5.72e-2) -	(3.30e-2) -	(3.71e-2) -	(2.32e-2) +	(2.68e-2) +	(1.78e-2) =	(5.74e-2) -	(4.00e-2) -	(2.96e-2)
WFG3	1.3030e-1	1.0920e-1	2.3262e-1	7.4264e-2	7.1527e-2	1.0408e-1	1.0323e-1	1.2861e-1	7.3253e-2
	(1.74e-2) -	(1.21e-2) -	(1.55e-2) -	(1.04e-2) =	(8.09e-3) =	(1.94e-2) -	(9.69e-3) -	(1.26e-2) -	(6.57e-3)
WFG4	2.2513e-1	1.8589e-1	4.2101e-1	1.4570e-1	1.4632e-1	2.6948e-1	2.0119e-1	2.2944e-1	1.3990e-1
	(2.18e-2) -	(1.32e-2) -	(2.84e-2) -	(1.42e-2) =	(1.77e-2) =	(1.05e-2) -	(2.33e-2) -	(2.07e-2) -	(1.42e-2)
WFG5	2.1080e-1	1.9681e-1	3.4803e-1	1.3632e-1	1.6203e-1	2.7662e-1	1.9086e-1	2.0763e-1	1.2692e-1
	(1.52e-2) -	(1.29e-2) -	(1.87e-2) -	(1.11e-2) -	(1.90e-2) -	(1.06e-2) -	(2.40e-2) -	(1.89e-2) -	(1.16e-2)
WFG6	2.2046e-1	1.8286e-1	3.9916e-1	1.4415e-1	1.4402e-1	2.7202e-1	1.9097e-1	2.1958e-1	1.3872e-1
	(2.40e-2) -	(1.07e-2) -	(3.92e-2) -	(1.25e-2) =	(1.21e-2) =	(1.16e-2) -	(2.27e-2) -	(2.18e-2) -	(1.48e-2)
WFG7	2.3641e-1	1.8760e-1	3.5488e-1	1.3882e-1	1.3314e-1	2.7062e-1	1.8527e-1	2.1450e-1	1.3757e-1
	(2.17e-2) -	(1.39e-2) -	(2.74e-2) -	(1.21e-2) =	(1.82e-2) =	(8.04e-3) -	(1.81e-2) -	(1.96e-2) -	(1.18e-2)
WFG8	2.3423e-1	1.7362e-1	4.3562e-1	1.3515e-1	1.6470e-1	2.4361e-1	2.3788e-1	2.1452e-1	1.3448e-1
	(2.50e-2) -	(1.55e-2) -	(2.22e-2) -	(9.01e-3) =	(1.89e-2) -	(1.09e-2) -	(1.69e-2) -	(1.96e-2) -	(1.03e-2)
WFG9	2.0218e-1	1.8625e-1	3.9492e-1	1.3823e-1	1.3404e-1	2.5747e-1	1.9071e-1	2.0282e-1	1.2876e-1
	(2.58e-2) -	(1.30e-2) -	(2.46e-2) -	(1.26e-2) -	(2.29e-2) =	(1.13e-2) -	(1.36e-2) -	(1.72e-2) -	(1.08e-2)
+/-/=	4/17/0	4/17/0	4/17/0	6/9/6	6/10/5	2/16/3	3/18/0	2/16/3	

Table S5. Spread values of EGC-CMOPSO and eight comparison algorithms.

Problem	NSGAIIARSBX	CAMOEA	MOEADCMA	PREA	Two_Arch2	GrEA	NMPSO	CMOPSO	EGCCMOPSO
ZDT1	3.5344e-1 (4.62e-2) -	2.5299e-1 (2.75e-2) -	2.9008e-1 (9.16e-4) -	2.3974e-1 (2.38e-2) -	3.3206e-1 (6.93e-3) -	9.9389e-1 (6.48e-2) -	1.4519e+0 (1.09e-1) -	1.3718e-1 (1.49e-2) =	1.3372e-1 (1.44e-2)
ZDT2	3.7117e-1 (5.25e-2) -	2.4924e-1 (2.13e-2) -	1.4288e-1 (7.21e-4) -	2.4845e-1 (2.77e-2) -	3.2954e-1 (6.97e-3) -	1.5790e+0 (5.00e-2) -	1.1871e+0 (8.67e-2) -	1.3351e-1 (1.28e-2) -	1.2584e-1 (1.32e-2)
ZDT3	4.0516e-1 (5.80e-2) -	2.7581e-1 (2.48e-2) -	7.1363e-1 (3.15e-2) -	2.7447e-1 (4.39e-2) -	1.8508e-1 (4.52e-2) -	1.3678e+0 (1.05e-1) -	1.7655e+0 (6.23e-3) -	1.5690e-1 (1.73e-2) =	1.5059e-1 (1.57e-2)
ZDT4	4.3259e-1 (6.95e-2) -	2.5554e-1 (2.35e-2) -	2.8920e-1 (2.05e-3) -	2.4188e-1 (2.91e-2) -	2.9500e-1 (2.65e-2) -	1.3694e+0 (4.19e-1) -	1.4427e+0 (9.65e-2) -	1.1646e-1 (1.53e-2) +	1.3511e-1 (1.42e-2)
ZDT6	4.3825e-1 (7.19e-2) -	2.6029e-1 (2.52e-2) -	1.6966e-1 (1.73e-1) +	2.2228e-1 (1.90e-2) -	2.4963e-1 (1.63e-1) -	1.8454e+0 (4.67e-2) -	4.8118e-1 (8.22e-2) -	5.3065e-1 (3.40e-1) -	1.9414e-1 (2.51e-1)
DTLZ1	4.4540e-1 (4.58e-2) -	2.1658e-1 (1.93e-2) =	2.3904e-2 (8.56e-3) +	1.5111e-1 (2.06e-2) +	1.8270e-1 (2.10e-2) +	5.8051e-1 (2.63e-1) -	3.3040e-1 (8.35e-2) -	5.3734e-1 (5.32e-2) -	2.3280e-1 (1.35e-1)
DTLZ2	4.6303e-1 (6.05e-2) -	2.1456e-1 (1.82e-2) -	2.3120e-1 (2.27e-2) -	1.5964e-1 (1.92e-2) -	2.1284e-1 (1.93e-2) -	1.0257e-1 (1.33e-2) -	3.2624e-1 (3.05e-2) -	3.9438e-1 (3.42e-2) -	9.4763e-2 (1.02e-2)
DTLZ3	7.0748e-1 (4.83e-1) -	5.2629e-1 (6.05e-1) -	5.0654e-1 (2.45e-1) =	1.6148e-1 (1.84e-2) +	3.5395e-1 (4.16e-1) +	6.1900e-1 (2.35e-1) -	3.2853e-1 (2.46e-2) +	6.1892e-1 (8.11e-2) -	4.3508e-1 (1.12e-1)
DTLZ4	4.6836e-1 (4.39e-2) -	2.0894e-1 (1.99e-2) -	5.7583e-1 (9.37e-2) -	4.3820e-1 (3.22e-1) -	2.0442e-1 (1.53e-1) -	1.1556e-1 (9.17e-2) =	3.1886e-1 (2.22e-2) -	4.6266e-1 (1.51e-1) -	9.9823e-2 (1.28e-2)
DTLZ5	4.2364e-1 (5.26e-2) -	2.9722e-1 (2.35e-2) -	1.8422e+0 (5.72e-2) -	2.3935e-1 (2.31e-2) -	3.7888e-1 (3.73e-2) -	1.6365e-1 (2.76e-2) =	1.0817e+0 (9.29e-2) -	3.9737e-1 (3.74e-2) -	1.6507e-1 (1.48e-2)
DTLZ6	5.8758e-1 (9.30e-2) -	2.8064e-1 (2.00e-2) -	2.0066e+0 (1.40e-2) -	2.5838e-1 (2.71e-2) -	4.9930e-1 (7.95e-3) -	4.7477e-1 (2.12e-1) -	1.0869e+0 (1.07e-1) -	4.2237e-1 (5.06e-2) -	1.7176e-1 (1.56e-2)
DTLZ7	5.0152e-1 (3.55e-2) -	3.3312e-1 (3.87e-2) -	1.2992e+0 (2.09e-2) -	3.2651e-1 (9.55e-2) -	2.1807e-1 (2.63e-2) +	1.1011e+0 (7.62e-2) -	5.6429e-1 (5.18e-2) -	5.1170e-1 (4.94e-2) -	2.4860e-1 (2.19e-2)
WFG1	5.8729e-1 (5.17e-2) -	3.5061e-1 (3.80e-2) -	9.6223e-1 (1.79e-1) -	2.7694e-1 (2.48e-2) +	3.4929e-1 (2.53e-2) -	1.3257e+0 (7.66e-2) -	1.5416e+0 (1.00e-1) -	6.9798e-1 (4.69e-2) -	3.3405e-1 (2.05e-2)
WFG2	5.0102e-1 (5.56e-2) -	3.3936e-1 (2.70e-2) -	7.0089e-1 (7.40e-2) -	2.9106e-1 (2.67e-2) -	2.7114e-1 (3.16e-2) =	9.0347e-1 (5.87e-2) -	1.4305e+0 (8.01e-2) -	4.9399e-1 (5.21e-2) -	2.6973e-1 (2.56e-2)
WFG3	6.0685e-1 (6.04e-2) -	3.0629e-1 (2.58e-2) -	1.1783e+0 (8.36e-2) -	2.2538e-1 (2.07e-2) -	1.9888e-1 (2.19e-2) =	1.2351e+0 (8.75e-2) -	4.3691e-1 (3.68e-2) -	5.7156e-1 (4.63e-2) -	1.9134e-1 (1.88e-2)
WFG4	4.4021e-1 (5.56e-2) -	2.9805e-1 (2.86e-2) -	8.5492e-1 (1.11e-1) -	2.3534e-1 (2.81e-2) -	2.0805e-1 (1.79e-2) =	4.9646e-1 (3.09e-2) -	3.2327e-1 (2.72e-2) -	4.3088e-1 (4.18e-2) -	2.1199e-1 (2.20e-2)
WFG5	4.5564e-1 (3.59e-2) -	3.1827e-1 (2.53e-2) -	5.4584e-1 (6.29e-2) -	2.2518e-1 (1.99e-2) -	2.2249e-1 (2.80e-2) =	5.2827e-1 (3.00e-2) -	3.1765e-1 (2.40e-2) -	4.2959e-1 (3.64e-2) -	2.1072e-1 (1.81e-2)
WFG6	4.2486e-1 (3.83e-2) -	2.9299e-1 (2.64e-2) -	6.5542e-1 (1.16e-1) -	2.4197e-1 (2.15e-2) -	2.2058e-1 (1.86e-2) =	5.2028e-1 (2.72e-2) -	3.4717e-1 (2.64e-2) -	4.0857e-1 (3.52e-2) -	2.1351e-1 (2.42e-2)
WFG7	4.7177e-1 (5.07e-2) -	3.0494e-1 (2.92e-2) -	5.2466e-1 (8.82e-2) -	2.3565e-1 (2.43e-2) -	2.0203e-1 (2.26e-2) =	5.0889e-1 (2.04e-2) -	3.2288e-1 (2.61e-2) -	4.0915e-1 (4.23e-2) -	2.0593e-1 (1.92e-2)
WFG8	4.6336e-1 (3.97e-2) -	2.7796e-1 (2.72e-2) -	6.4394e-1 (1.07e-1) -	2.2866e-1 (1.65e-2) -	2.3218e-1 (2.36e-2) -	5.3331e-1 (3.76e-2) -	4.2223e-1 (2.51e-2) -	4.1663e-1 (3.57e-2) -	2.0581e-1 (1.99e-2)
WFG9	4.3153e-1 (5.22e-2) -	3.1563e-1 (2.33e-2) -	6.7149e-1 (1.03e-1) -	2.2726e-1 (2.22e-2) -	1.9670e-1 (2.37e-2) +	4.7705e-1 (2.88e-2) -	3.2664e-1 (2.41e-2) -	4.1622e-1 (3.71e-2) -	2.1131e-1 (1.72e-2)
+/-/=	0/21/0	0/20/1	2/18/1	3/18/0	4/11/6	0/19/2	1/20/0	1/18/2	

Table S6. Time required for 50 iterations of EGC-CMOPSO and eight comparison algorithms.

Problem	NSGAIIARSBX	CAMOEA	MOEADCMA	PREA	Two_Arch2	GrEA	NMPSO	CMOPSO	EGCCMOPSO
ZDT3	1.7092e-1	1.9691e-1	8.7570e-1	1.9492e-1	4.1180e-1	2.1751e-1	9.9085e-1	2.8260e-1	9.1459e-1
	(1.15e-2) +	(8.57e-3) +	(2.06e-1) +	(1.77e-2) +	(3.88e-2) +	(5.22e-2) +	(1.12e-1) -	(3.92e-2) +	(4.33e-2)
DTLZ4	1.6357e-1	2.0613e-1	1.0765e+0	2.3533e-1	6.9375e-1	5.3377e-1	1.2421e+0	6.9042e-1	6.8516e-1
	(1.18e-2) +	(2.01e-2) +	(1.43e-1) -	(3.61e-2) +	(1.20e-1) =	(1.02e-1) +	(1.20e-1) -	(1.85e-1) =	(5.01e-2)
DTLZ5	1.6869e-1	1.9849e-1	1.1252e+0	2.8921e-1	6.7909e-1	4.4409e-1	1.0653e+0	7.0372e-1	6.9291e-1
	(1.71e-2) +	(1.35e-2) +	(1.15e-1) -	(2.72e-2) +	(3.97e-2) =	(2.41e-2) +	(8.18e-2) -	(6.36e-2) =	(3.57e-2)
WFG2	1.7013e-1	2.1880e-1	1.2991e+0	2.4662e-1	8.0565e-1	4.7734e-1	1.0622e+0	4.5692e-1	7.4607e-1
	(1.98e-2) +	(1.40e-2) +	(1.51e-1) -	(1.87e-2) +	(6.00e-2) -	(5.75e-2) +	(9.07e-2) -	(3.08e-2) +	(3.12e-2)
WFG3	1.7709e-1	2.3877e-1	1.2967e+0	3.8766e-1	1.1930e+0	5.6626e-1	1.5613e+0	7.2360e-1	9.6898e-1
	(1.03e-2) +	(2.39e-2) +	(9.23e-2) -	(2.87e-2) +	(8.19e-2) -	(2.05e-2) +	(1.03e-1) -	(5.23e-2) +	(4.53e-2)
WFG4	1.7376e-1	2.3357e-1	1.2509e+0	3.3081e-1	1.2004e+0	4.8516e-1	1.4127e+0	1.4456e+0	1.0742e+0
	(1.30e-2) +	(2.23e-2) +	(1.18e-1) -	(3.13e-2) +	(4.33e-2) -	(3.03e-2) +	(7.72e-2) -	(8.13e-2) -	(4.40e-2)
+/-/=	6/0/0	6/0/0	1/5/0	6/0/0	1/3/2	6/0/0	0/6/0	3/1/2	

Supplementary Figure

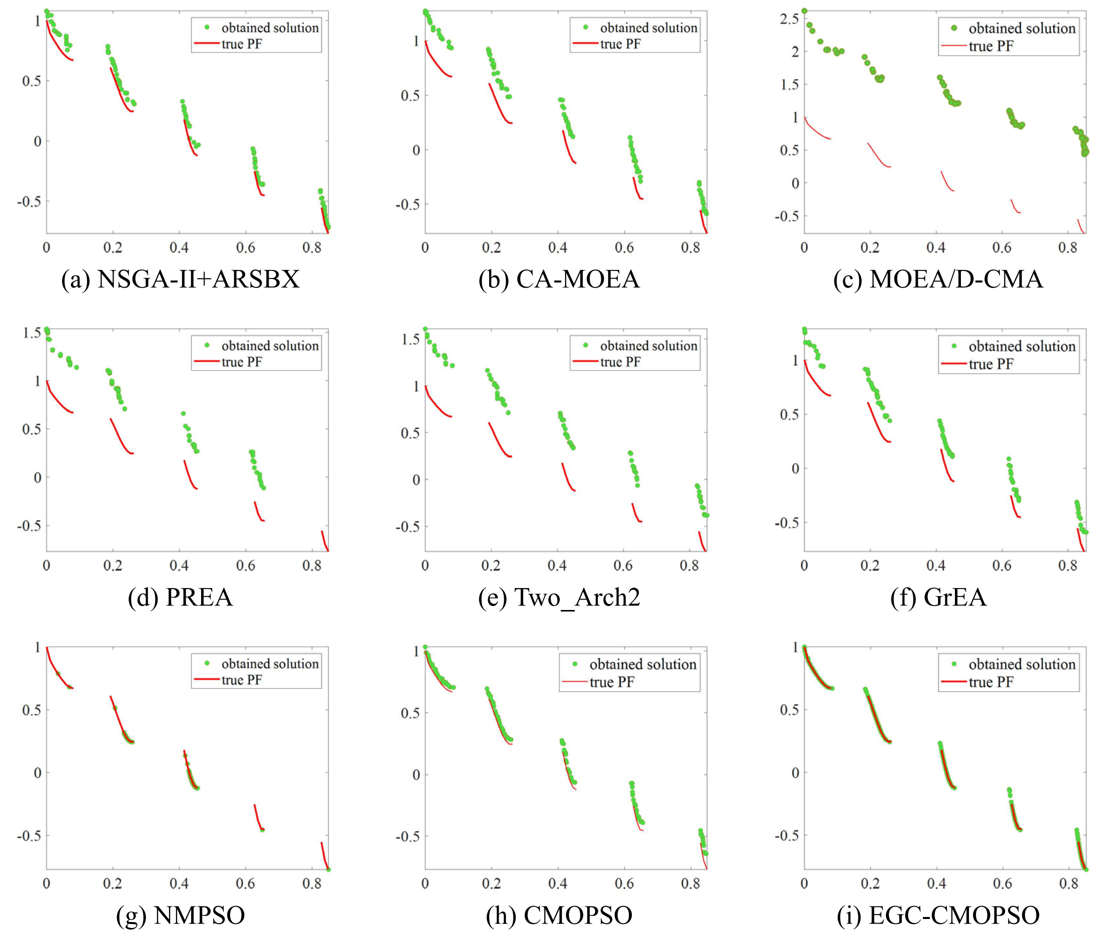

Figure S1. The non-dominated solutions obtained by EGC-CMOPSO and eight comparison algorithms on two-objective ZDT3.

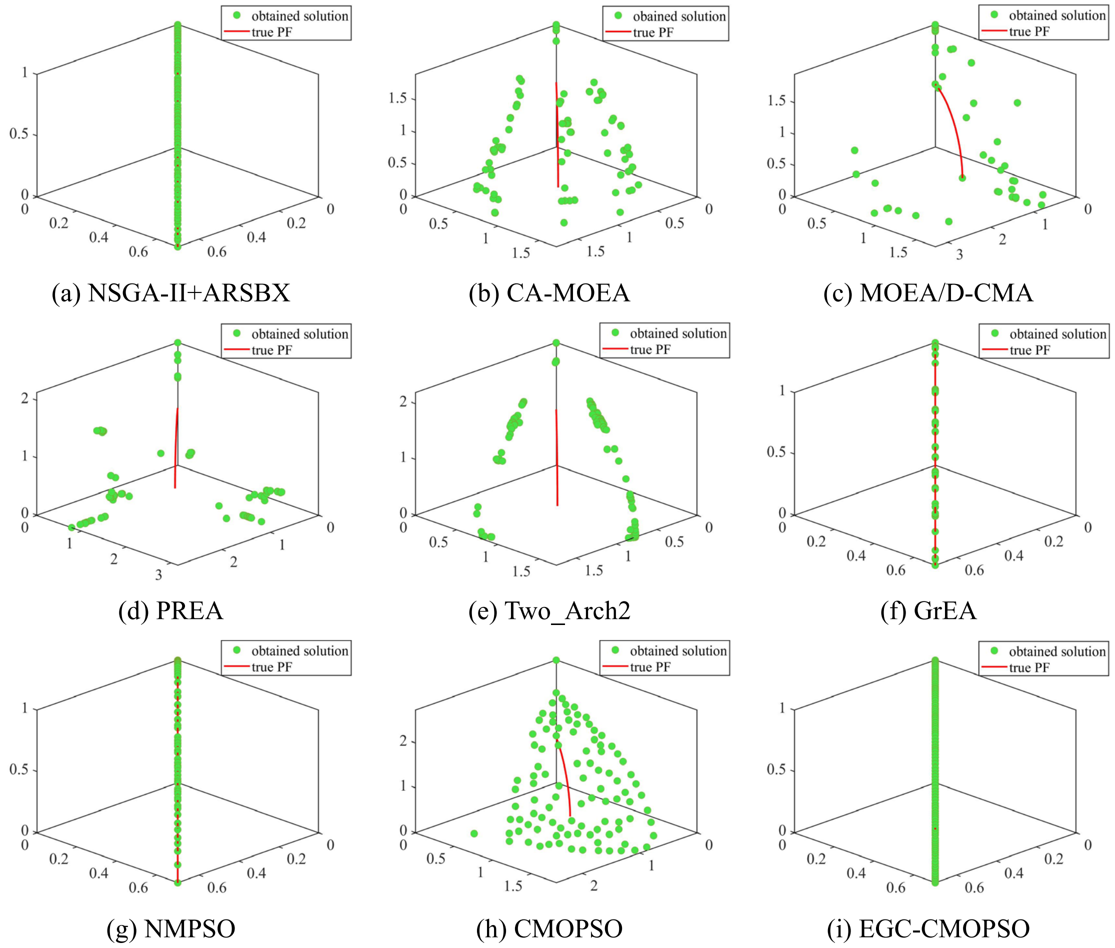

Figure S2. The non-dominated solutions obtained by EGC-CMOPSO and eight comparison algorithms on three-objective DTLZ6.

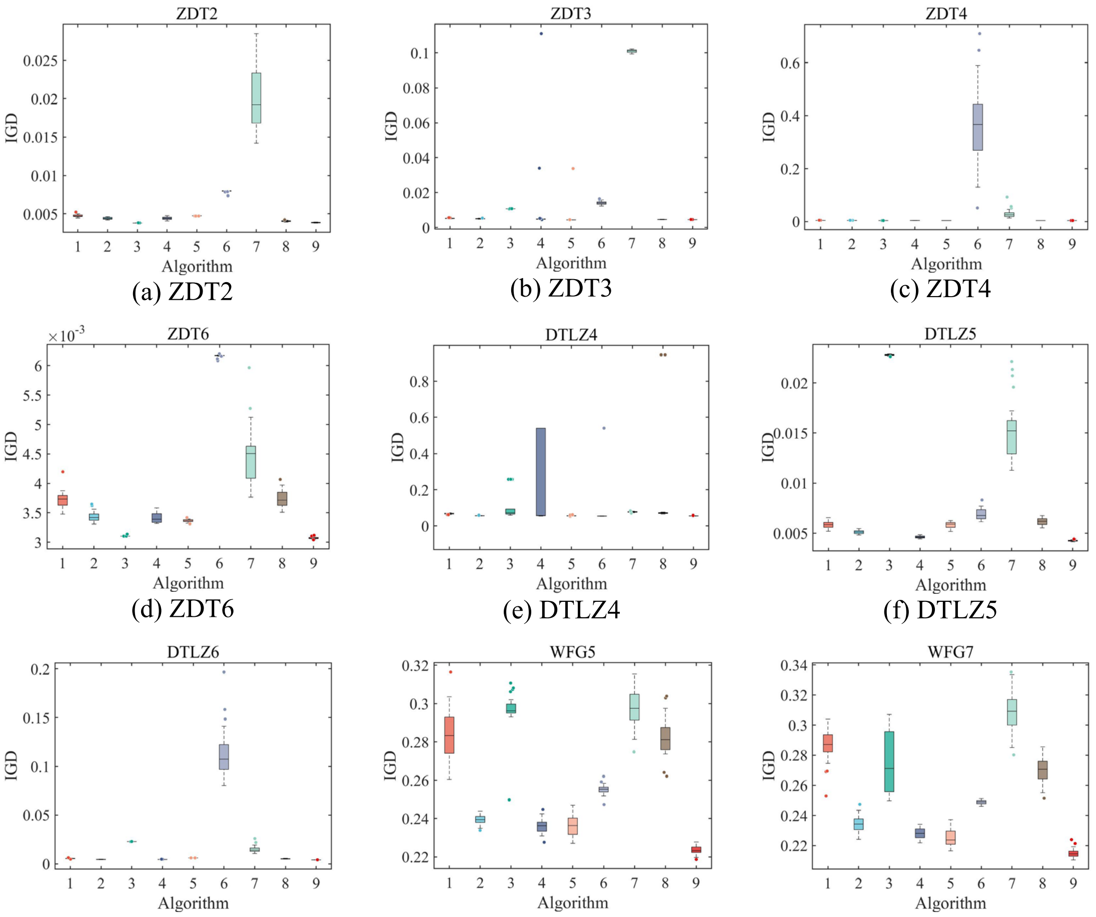

(a) ZDT2 (b) ZDT3 (c) ZDT4
(d) ZDT6 (e) DTLZ4 (f) DTLZ5
(g) DTLZ6 (h) WFG5 (i) WFG7

Figure S3. The performance of EGC-CMOPSO and eight comparison algorithms for IGD on 9 test problems among 30 runs when N =100. The values on the x-coordinate denote the following algorithms: 1=NSGA-II+ARSBX, 2=CA-MOEA, 3=MOEA/D-CMA, 4=PREA, 5=Two_Arch2, 6=GrEA, 7=NMPSO, 8=CMOPSO, 9=EGC-CMOPSO

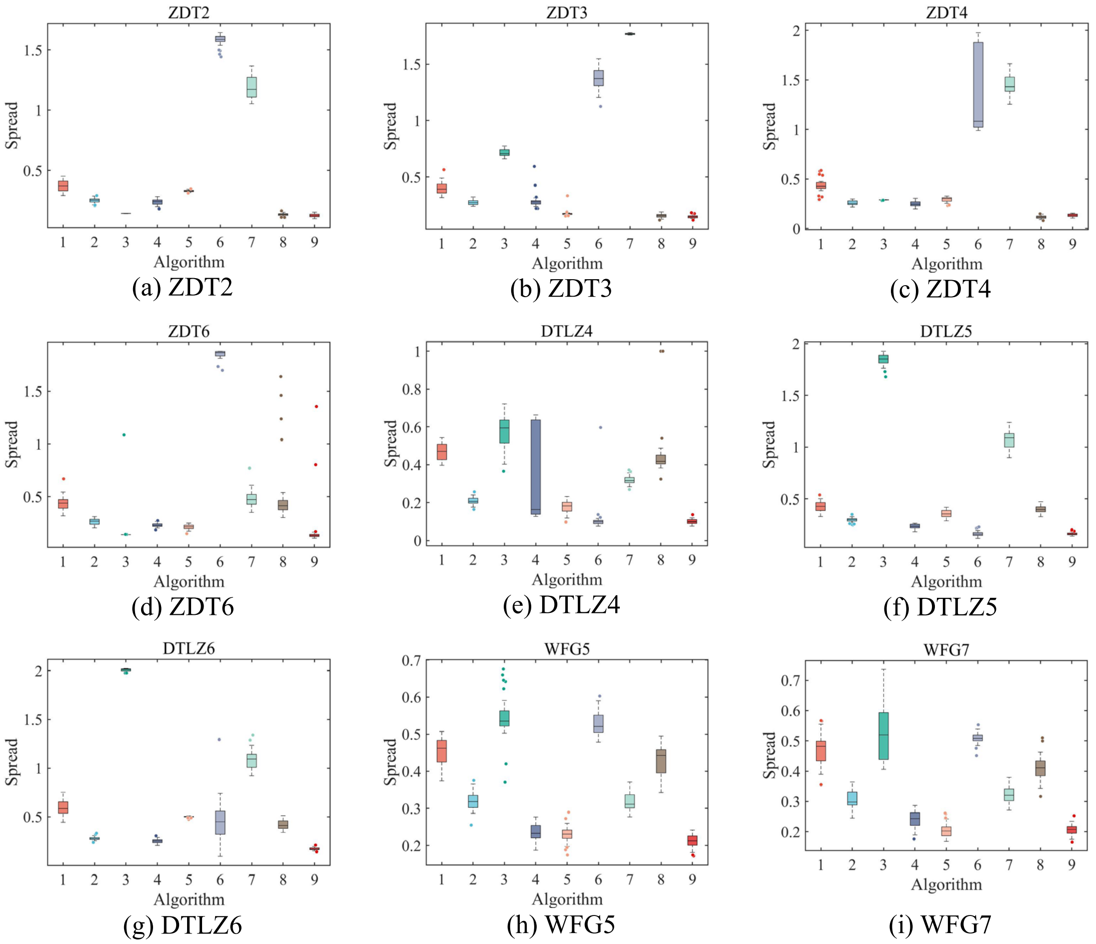

Figure S4. The performance of EGC-CMOPSO and eight comparison algorithms for Spread on 9 test problems among 30 runs when N =100. The values on the x-coordinate denote the following algorithms: 1=NSGA-II+ARSBX, 2=CA-MOEA, 3=MOEA/D-CMA, 4=PREA, 5=Two_Arch2, 6=GrEA, 7=NMPSO, 8=CMOPSO, 9=EGC-CMOPSO

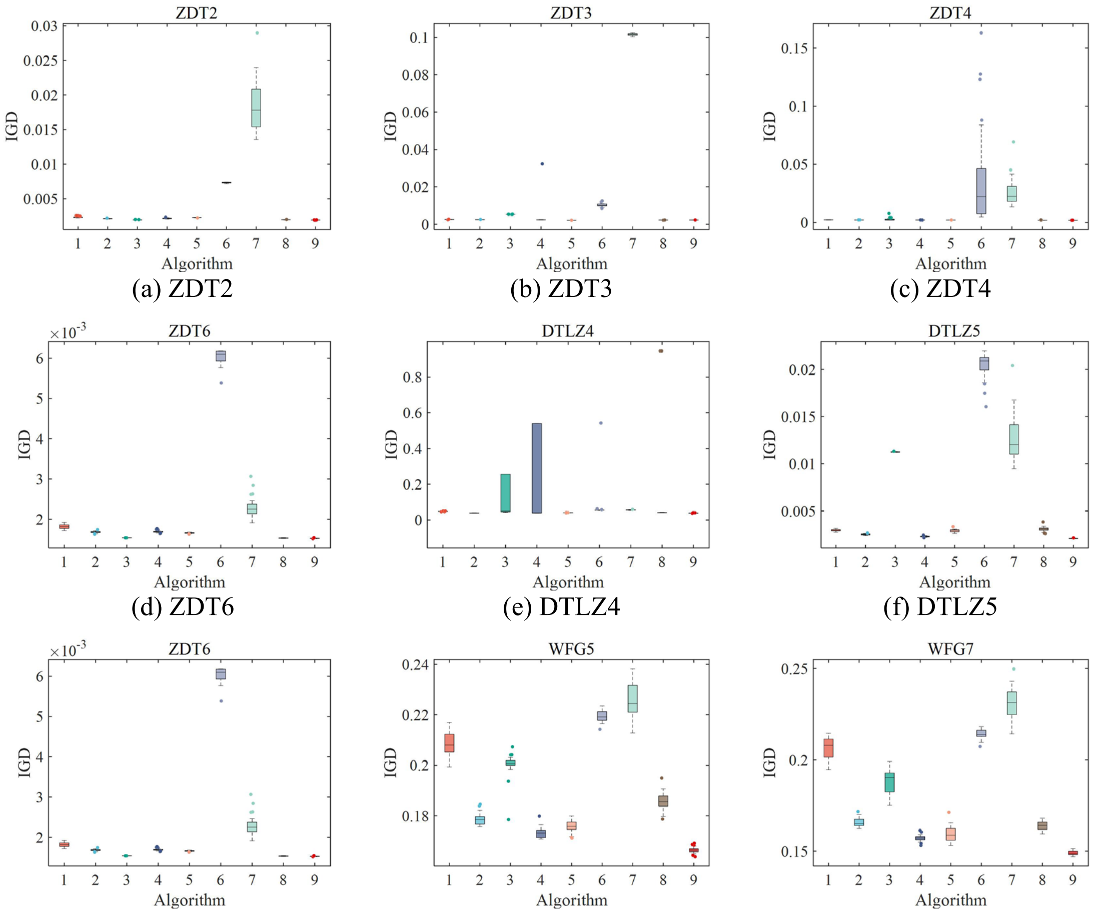

(a) ZDT2 (b) ZDT3 (c) ZDT4
(d) ZDT6 (e) DTLZ4 (f) DTLZ5
(g) DTLZ6 (h) WFG5 (i) WFG7

Figure S5. The performance of EGC-CMOPSO and eight comparison algorithms for IGD on 9 test problems among 30 runs when N =200. The values on the x-coordinate denote the following algorithms: 1=NSGA-II+ARSBX, 2=CA-MOEA, 3=MOEA/D-CMA, 4=PREA, 5=Two_Arch2, 6=GrEA, 7=NMPSO, 8=CMOPSO, 9=EGC-CMOPSO

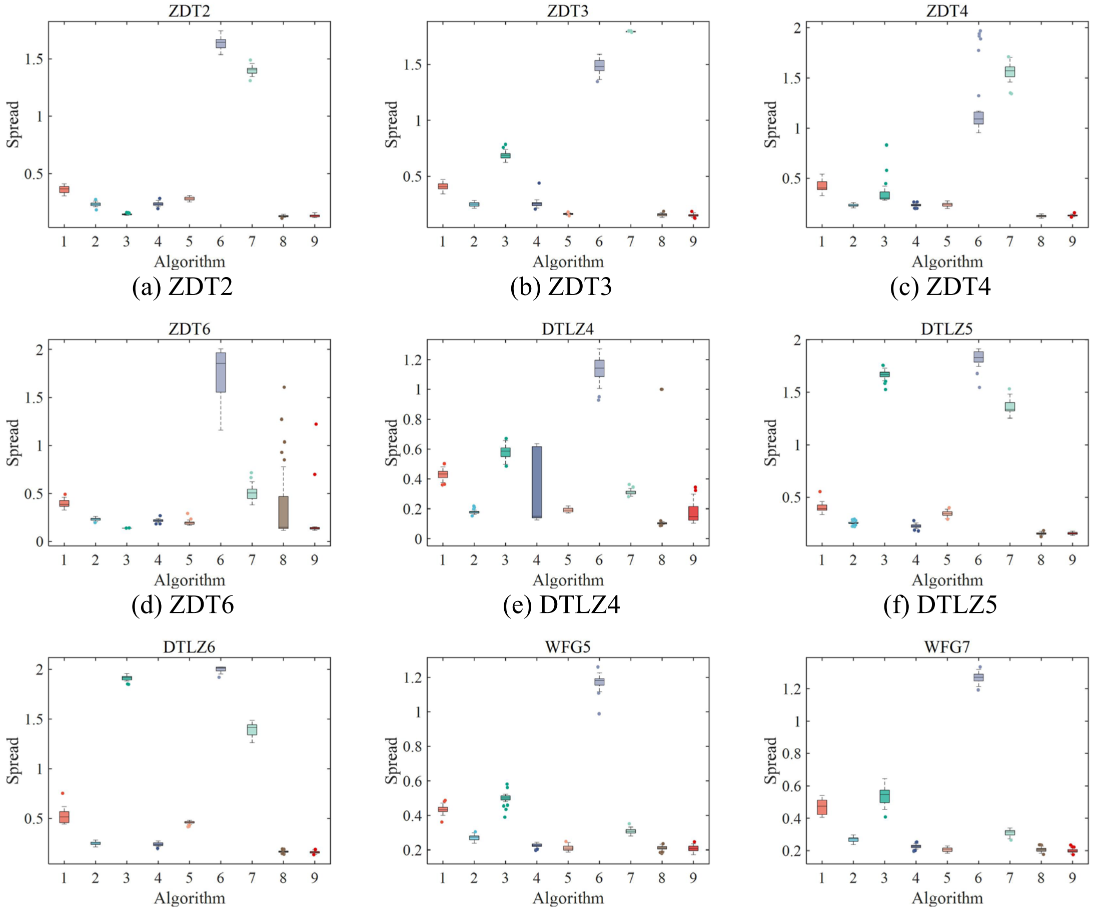

(a) ZDT2 (b) ZDT3 (c) ZDT4

(d) ZDT6 (e) DTLZ4 (f) DTLZ5

(g) DTLZ6 (h) WFG5 (i) WFG7

Figure S6. The performance of EGC-CMOPSO and eight comparison algorithms for Spread on 9 test problems among 30 runs when N =200. The values on the x-coordinate denote the following algorithms: 1=NSGA-II+ARSBX, 2=CA-MOEA, 3=MOEA/D-CMA, 4=PREA, 5=Two_Arch2, 6=GrEA, 7=NMPSO, 8=CMOPSO, 9=EGC-CMOPSO

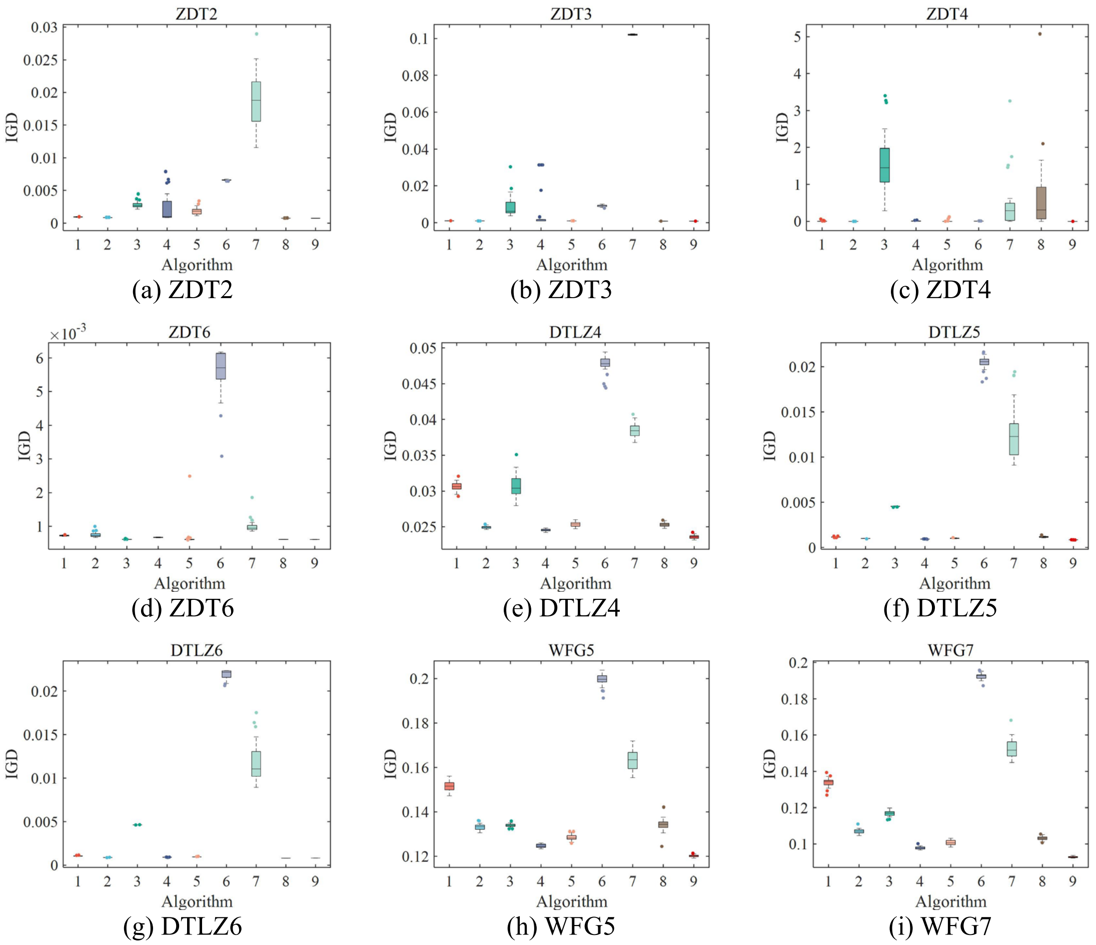

Figure S7. The performance of EGC-CMOPSO and eight comparison algorithms for IGD on 9 test problems among 30 runs when N =500. The values on the x-coordinate denote the following algorithms: 1=NSGA-II+ARSBX, 2=CA-MOEA, 3=MOEA/D-CMA, 4=PREA, 5=Two_Arch2, 6=GrEA, 7=NMPSO, 8=CMOPSO, 9=EGC-CMOPSO

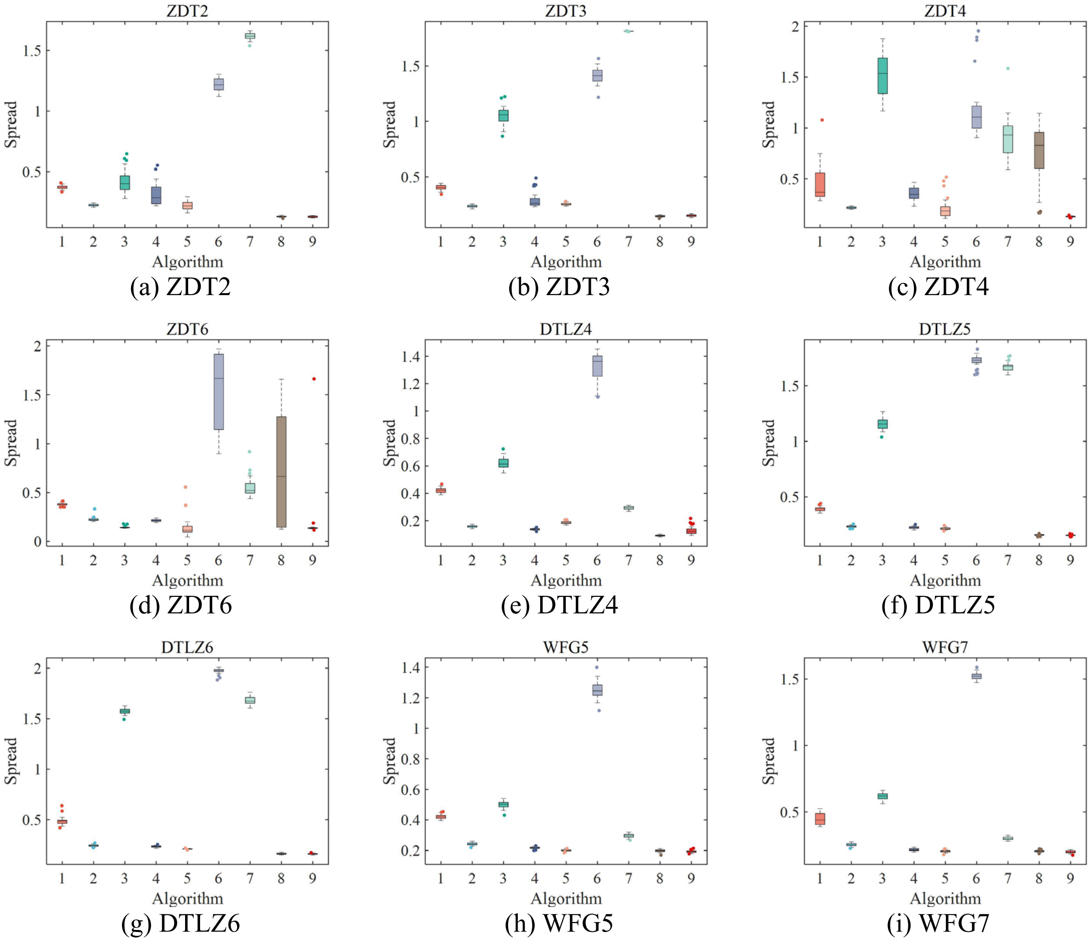

Figure S8. The performance of EGC-CMOPSO and eight comparison algorithms for Spread on 9 test problems among 30 runs when N =500. The values on the x-coordinate denote the following algorithms: 1=NSGA-II+ARSBX, 2=CA-MOEA, 3=MOEA/D-CMA, 4=PREA, 5=Two_Arch2, 6=GrEA, 7=NMPSO, 8=CMOPSO, 9=EGC-CMOPSO

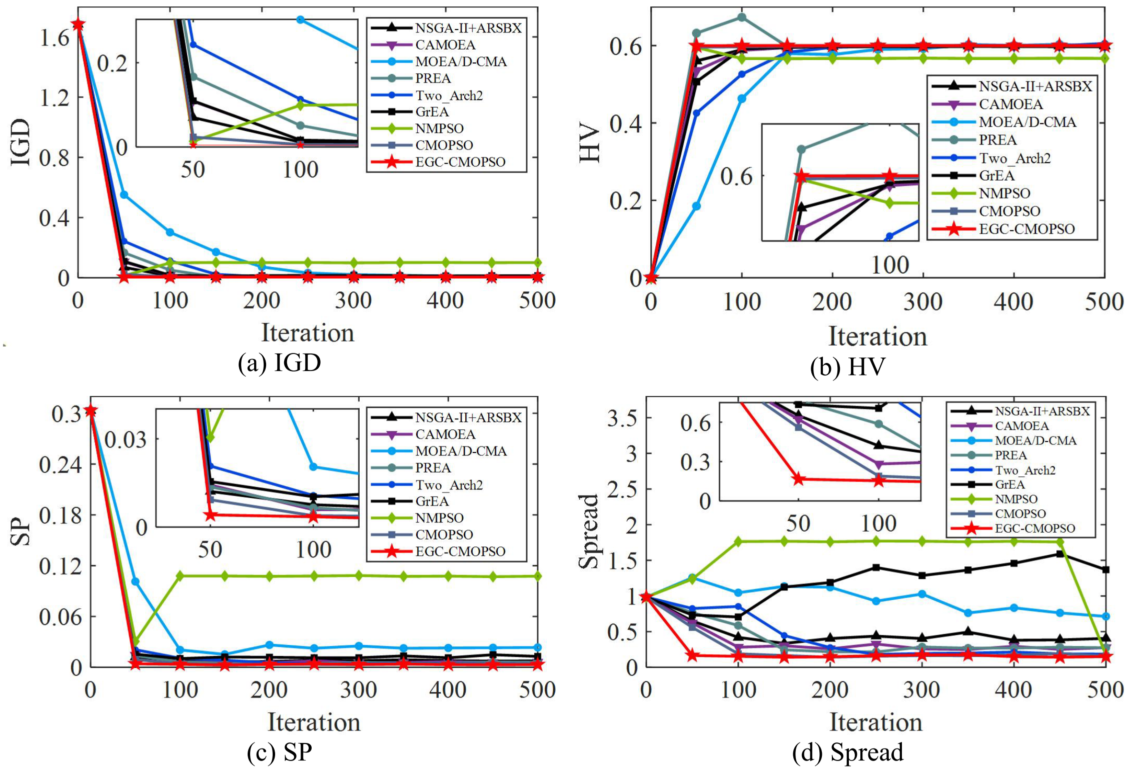

Figure S9. The performance metrics of EGC-CMOPSO and eight comparison algorithms on two-objective ZDT3, averaging over 30 runs.

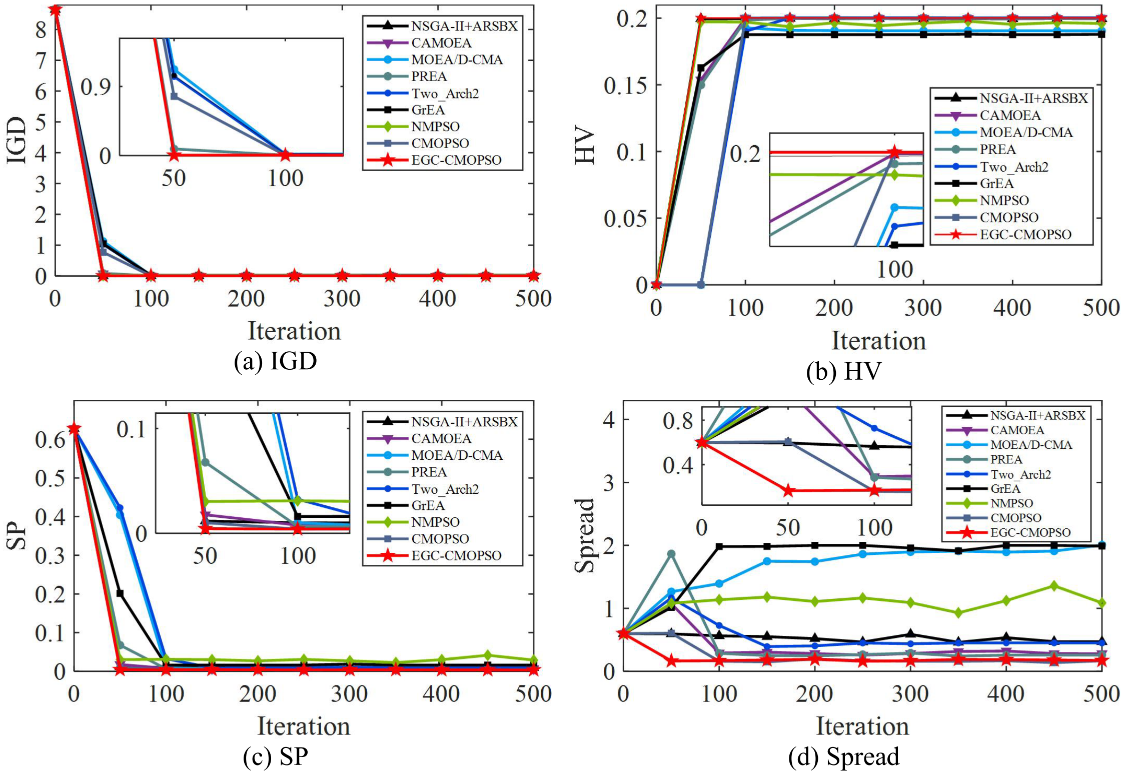

Figure S10. The performance metrics of EGC-CMOPSO and eight comparison algorithms on three-objective DTLZ6, averaging over 30 runs.

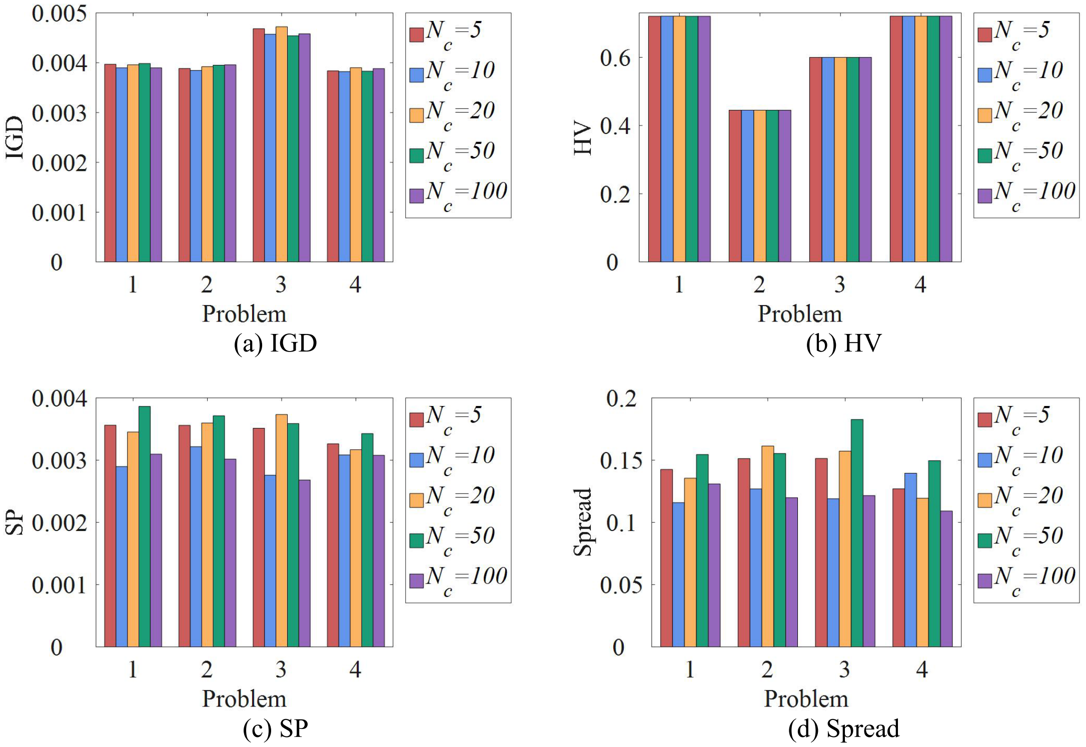

Figure S11. The performance metrics of EGC-CMOPSO with the different numbers of clusters on 4 two-objective test problems. The values on the x-coordinate denote the following algorithms: 1=ZDT1, 2=ZDT2, 3=ZDT3, 4=ZDT4

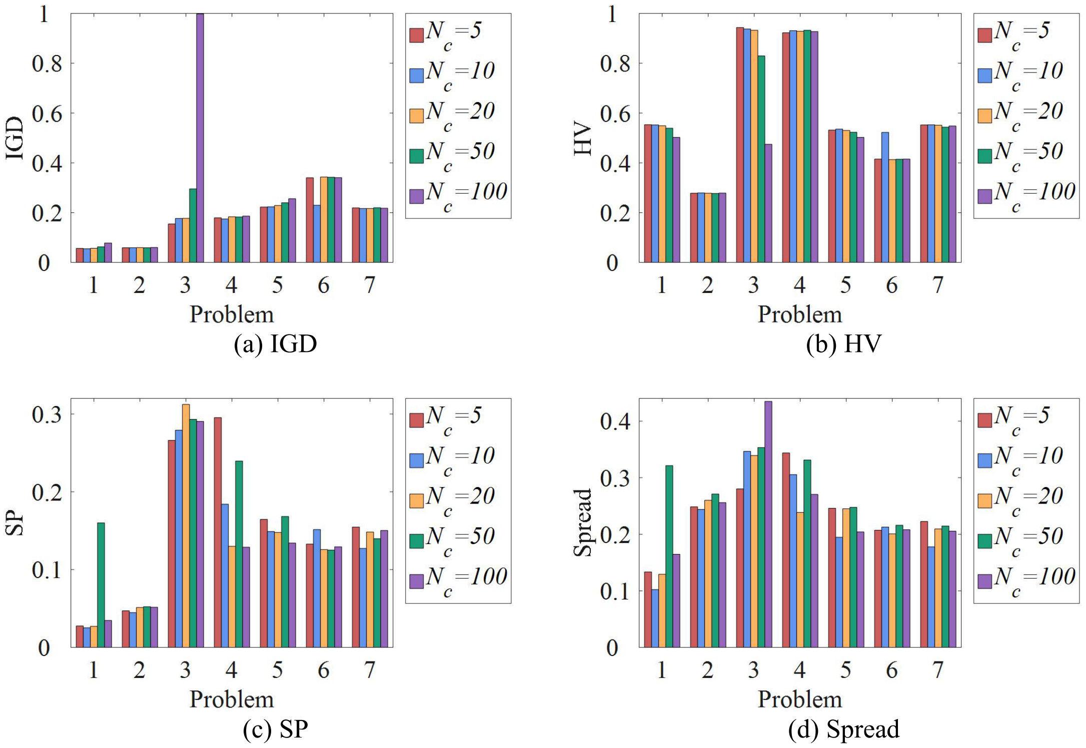

Figure S12. The performance metrics of EGC-CMOPSO with the different numbers of clusters on 7 three-objective test problems. The values on the x-coordinate denote the following algorithms: 1= DTLZ2, 2= DTLZ7, 3= WFG1, 4= WFG2, 5= WFG4, 6= WFG6, 7= WFG7
